# Global, regional, and national burden of age-related hearing loss from 1990 to 2019

**DOI:** 10.18632/aging.203782

**Published:** 2021-12-15

**Authors:** Jinyu Man, Hui Chen, Tongchao Zhang, Xiaolin Yin, Xiaorong Yang, Ming Lu

**Affiliations:** 1 School of Public Health, Cheeloo College of Medicine, Shandong University, Jinan, China; 2 Clinical Epidemiology Unit, Qilu Hospital of Shandong University, Jinan, China; 3 Clinical Research Center of Shandong University, Qilu Hospital, Cheeloo College of Medicine, Shandong University, Jinan, China

**Keywords:** age-related hearing loss, global burden of disease, DALYs, prevalent cases, temporal trend

## Abstract

The global distribution and temporal trend of age-related hearing loss (ARHL) are unknown, and we aimed to investigate magnitudes and temporal trends of ARHL burden and its influencing factors at the national, regional, and global levels. Based on the information of Global Burden of Disease Study 2019, we calculated the estimated annual percentage change to quantify the global, regional, and national temporal trends of age-standardized rates (ASRs) of ARHL by gender, age, and severity. The number of prevalent cases and disability-adjusted life years (DALYs) of ARHL increased from 751.50 million and 22.01 million in 1990 to 1456.66 million and 40.24 million in 2019, respectively. Except for a few countries such as Niger and Burkina Faso, the age-standardized prevalence rate and age-standardized DALYs rate showed a downward trend in most countries and regions. Mild ARHL accounted for the largest proportion in all ARHL, and only mild ARHL showed an upward trend in ASRs. In most regions, the proportion of ARHL disease burden attributable to occupational noise showed a downward trend in the past 30 years. In 2019, ARHL disease burden attributable to occupational noise declined with the increase of socio-demographic index in countries. Although the ASR of ARHL in most parts of the world is declining, the absolute disease burden of ARHL is still heavy. Understanding the real-time disease burden of ARHL and its temporal trend is of great significance for formulating more effective preventive measures and reducing the ARHL burden.

## INTRODUCTION

Hearing loss is a major global health problem concerned by WHO. In 2019, 1570 million people were affected by various degrees of hearing loss, roughly equivalent to one out of every five people with this disease [1]. Age-related hearing loss (ARHL) is a frequently reported chronic health problem in the elderly, accounting for the vast majority of hearing loss [2]. In addition to the impact on communication ability, ARHL can also lead to lots of serious health problems, including mental health problems, Alzheimer’s disease, and so on [3]. A full understanding of the temporal trends and its influencing factors of ARHL disease burden is the basis for formulating targeted public policies and reducing the disease burden of the population.

However, most of the current studies only describe the prevalence rate of hearing loss in a country or a region, and few studies describe the global temporal trends and influential factors of the temporal trends of ARHL. The Global Burden of Disease (GBD) study 2019 hearing loss collaborators explored and fully reported the global prevalence of hearing loss from 1990 to 2019 and predicted the prevalence of hearing loss by 2050, which provided a basis for the prevention and control of hearing loss [1]. Marcin Masalski et al. conducted a cross-sectional study using the APP Hearing Test to assess the prevalence of hearing loss in 74 countries [4]. Stevens et al. found that rates of hearing loss in children and adults are much higher in low and middle-income countries than in high-income countries using data from 42 studies carried out between 1970 and 2010 in 29 countries [5]. Occupational noise is also an important factor affecting the incidence of ARHL [3]. However, the temporal trends of ARHL burden and the influence of factors, such as occupational noise, on temporal trends of ARHL disease burden are still unknown. Under the premise of increasing population aging, understanding the temporal trends and its influencing factors of the ARHL disease burden is essential to prioritize effective preventive measures.

GBD study 2019 systematically assesses and updates the disease burden and influencing factors in 204 countries and territories, which provides a unique opportunity to research temporal trends and its influential factors of ARHL disease burden. In this study, we aim to estimate the temporal trends and its influential factors of ARHL disease burden by genders, age groups, and hearing loss severity at global, regional and national levels. Our findings will provide the basis for the allocation of medical resources and policymaking for the reduction of the ARHL disease burden globally.

## RESULTS

### The global burden due to ARHL

**Figure 1:**
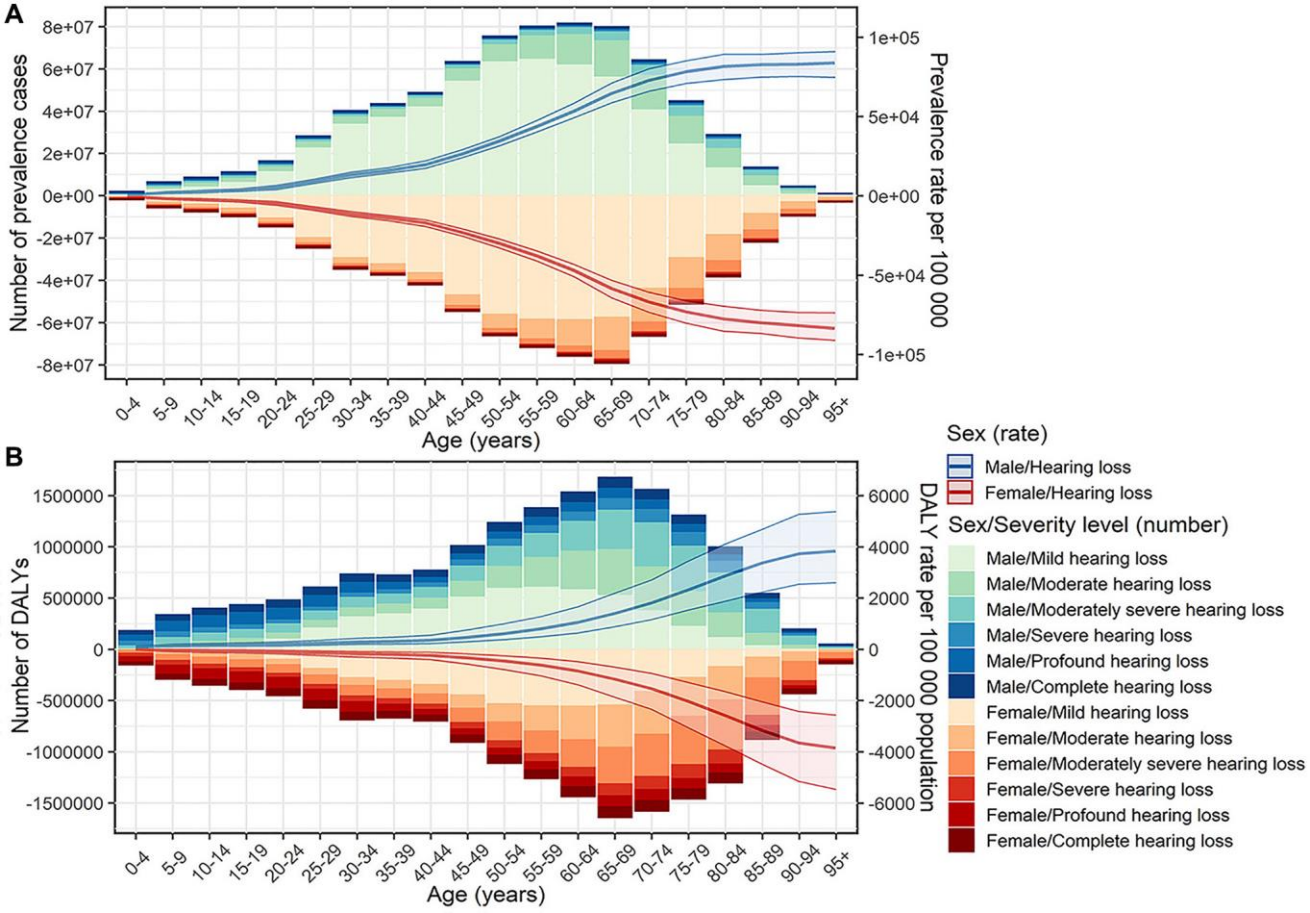
**Prevalent cases, DALYs and the corresponding rates of age-related hearing loss by sex, age group, and severity in 2019.** (**A**) Prevalent cases and prevalence rate; (**B**) DALYs and DALY rate. Abbreviation: DALY: disability-adjusted life year.

Globally, the ARHL patients increased from 751.50 [95% uncertainty interval (UI): 716.45, 787.33] million in 1990 to 1456.66 (95% UI: 1395.61, 1519.30) million in 2019, and the age-standardized prevalence rate (ASPR) showed a slight upward trend from 17.33 to 17.76 per 100 during the period, with an estimated annual percentage change (EAPC) of 0.08 [95% confidence interval (CI): 0.07, 0.10]. The estimated disability-adjusted life years (DALYs) of ARHL worldwide increased from 22.01 (95% UI: 14.91, 31.34) million to 40.24 (95% UI: 27.39, 57.13) million over the past 30 years, with a stable age-standardized DALY rate (ASDR) of about 0.005. The number of males with ARHL was 742.04 (95% UI 711.33, 774.94) million in 2019, accounting for 50.9% of all ARHL patients, causing 20.17 (95% UI 13.71, 28.75) million DALYs. Although women had lower ASPR and ASDR than men, female ASPR rose faster and ASDR fell more slowly (Table 1). Most patients were between 45–74 years old, and the burden of disease in this age group was also heavier (Figure 1).

**Table 1:** Age-related hearing loss prevalent cases and burden in 1990 and 2019 and the temporal trends from 1990 to 2019.

**Characteristics**	**1990**				**2019**				**EAPC (1990–2019)**	
	**Prevalent cases**		**DALYs**		**Prevalent cases**		**DALYs**		**ASPR**	**ASDR**
		**ASPR**		**ASDR**		**ASPR**		**ASDR**		
	**No. × 10^6^** **(95% UI)**	**No. × 10^−2^** **(95% UI)**	**No. × 10^6^** **(95% UI)**	**No. × 10^−3^** **(95% UI)**	**No. × 10^6^** **(95% UI)**	**No. × 10^−2^** **(95% UI)**	**No. × 10^6^** **(95% UI)**	**No. × 10^−3^** **(95% UI)**	**No.** **(95% CI)**	**No.** **(95% CI)**
**Global**	751.50 (716.45, 787.33)	17.33 (16.58, 18.10)	22.01 (14.91, 31.34)	5.09 (3.47, 7.25)	1456.66 (1395.61, 1519.30)	17.76 (17.01, 18.52)	40.24 (27.39, 57.13)	4.99 (3.40, 7.10)	0.08 (0.07, 0.10)^*^	−0.07 (−0.09,-0.05)
**Sex**										
Males	386.11 (367.62, 405.17)	18.49 (17.68, 19.33)	11.11 (7.44, 15.88)	5.40 (3.67, 7.70)	742.04 (711.33, 774.94)	18.74 (17.98, 19.56)	20.17 (13.71, 28.75)	5.24 (3.57, 7.46)	0.05 (0.04, 0.06)*	−0.10 (−0.12, −0.08)
Females	365.39 (348.73, 381.94)	16.24 (15.53, 16.95)	10.90 (7.47, 15.46)	4.80 (3.29, 6.82)	714.62 (685.19, 745.32)	16.81 (16.11, 17.52)	20.07 (13.64, 28.40)	4.76 (3.24, 6.76)	0.12 (0.11,0.13)*	−0.05 (−0.07, −0.03)
**Severity**										
Mild hearing loss	553.35 (523.49, 583.60)	12.48 (11.83, 13.10)	5.25 (2.34, 10.09)	0.12 (0.05, .23)	1083.06 (1029.88, 1136.29)	13.09 (12.44, 13.74)	10.28 (4.57, 19.82)	0.12 (0.06, 0.24)	0.18 (0.17, 0.19)^*^	0.18 (0.17, 0.19)^*^
Moderate hearing loss	126.66 (109.55, 144.62)	3.14 (2.75, 3.56)	3.32 (1.90, 5.36)	0.08 (0.05, 0.13)	245.98 (214.76, 278.22)	3.05 (2.67, 3.44)	6.45 (3.70, 10.47)	0.08 (0.05, 0.13)	-0.17 (-0.21,-0.13)	−0.17 (−0.21, −0.12)
Moderately severe hearing loss	43.24 (35.89, 51.86)	1.09 (0.92, 1.31)	3.91 (2.53, 5.73)	0.10 (0.06, 0.14)	82.75 (69.07, 99.54)	1.05 (0.88, 1.25)	7.47 (4.86, 10.89)	0.09 (0.06, 0.14)	-0.11 (-0.14,-0.09)	−0.11 (−0.13, −0.08)
Severe hearing loss	9.86 (7.24, 12.73)	0.23 (0.18, 0.30)	1.52 (0.90, 2.31)	0.04 (0.02, 0.05)	17.43 (13.40, 22.10)	0.22 (0.17, 0.28)	2.69 (1.62, 4.02)	0.03 (0.02, 0.05)	−0.18 (−0.22, −0.13)	−0.17 (−0.21, −0.13)
Profound hearing loss	10.52 (7.97, 13.57)	0.21 (0.17, 0.27)	2.11 (1.29,3.22)	0.04 (0.03, 0.06)	15.89 (12.36,20.10)	0.20 (0.16, 0.26)	3.18 (2.00, 4.73)	0.04 (0.03, 0.06)	−0.20 (−0.23, −0.17)	−0.19 (−0.22, −0.17)
Complete hearing loss	7.87 (5.88, 10.03)	0.17 (0.13, 0.22)	1.66 (0.99, 2.52)	0.04 (0.02, 0.05)	11.54 (9.05, 14.35)	0.15 (0.12, 0.18)	2.42 (1.52, 3.65)	0.03 (0.02, 0.05)	−0.59 (−0.62, −0.56)	−0.59 (−0.62, −0.56)
**SDI region**										
High	128.53 (121.99, 135.28)	12.90 (12.27, 13.56)	3.69 (2.52, 5.26)	3.70 (2.52, 5.28)	212.17 (201.03, 223.56)	12.99 (12.35, 13.64)	6.09 (4.14, 8.64)	3.60 (2.45, 5.16)	0.01 (0.00,0.02)*	−0.07 (−0.08, −0.06)
High-middle	190.15 (181.89, 198.78)	17.18 (16.44, 17.94)	5.29 (3.58, 7.55)	4.90 (3.32, 6.99)	338.17 (324.11, 352.67)	17.70 (16.97, 18.45)	8.93 (6.04,12.80)	4.78 (3.26, 6.80)	0.12 (0.10, 0.13)*	−0.08 (−0.11, −0.06)
Middle	239.59 (228.42,251.13)	19.45 (18.63, 20.27)	6.97 (4.68, 9.94)	5.69 (3.90, 8.09)	498.57 (478.56, 519.76)	19.37 (18.60, 20.15)	13.23 (8.95, 18.86)	5.36 (3.67, 7.62)	0.00 (−0.02, 0.01)	−0.21 (−0.23, −0.18)
Low-middle	138.84 (131.59, 146.73)	19.05 (18.22, 19.92)	4.25 (2.86, 6.02)	5.74 (3.96, 8.13)	287.32 (274.41, 300.34)	18.98 (18.21, 19.82)	8.15 (5.54, 11.62)	5.47 (3.74, 7.74)	−0.02 (−0.03, 0.00)	−0.21 (−0.24,-0.18)
Low	53.98 (50.78, 57.46)	17.69 (16.91, 18.51)	1.80 (1.21, 2.53)	5.62 (3.86, 7.93)	119.65 (112.82, 127.05)	17.53 (16.80, 18.30)	3.82 (2.58, 5.40)	5.37 (3.71, 7.59)	−0.03 (−0.04, −0.02)	−0.18 (−0.21, −0.16)

### Variation in ARHL burden at the national and regional level

**Figure 2:**
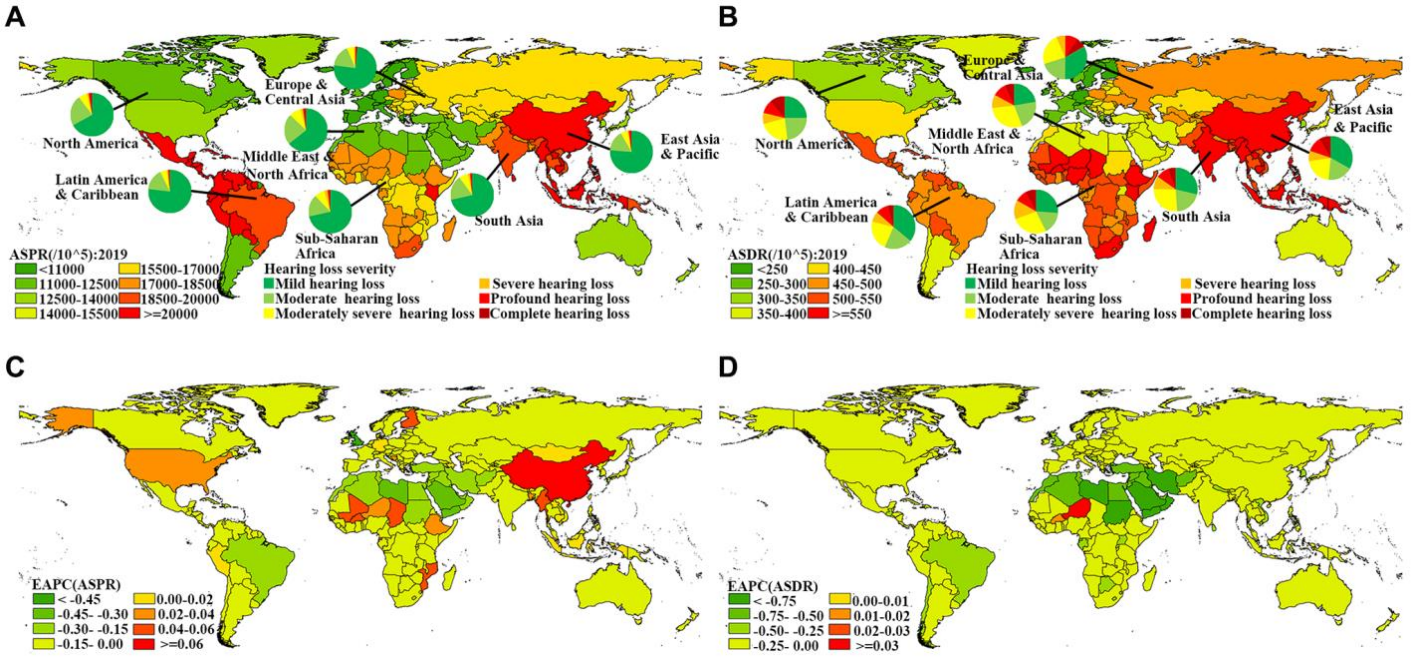
**Global distribution of ASRs and the corresponding EAPCs in age-related hearing loss.** (**A**) ASPR in 2019; (**B**) ASDR in 2019; (**C**) EAPC of ASPR from 1990 to 2019; (**D**) EAPC of ASDR from 1990 to 2019. Abbreviations: ASR: age-standardized rate; EAPC: estimated annual percentage change; ASPR: age-standardized prevalence rate; ASDR: age-standardized DALY rate; DALY: disability-adjusted life year.

The global variety of ASPR and ASDR of ARHL was around 2.4 and 3.5 times in 2019, respectively, with the highest ASPR (21.35/100) and ASDR (6.77/1000) in Kenya, and the lowest ASPR (8.75/100) and ASDR (1.96/1000) in Sweden. Overall, the ASPR in 2019 was higher than 17/100 in 98 countries and territories, including China, India, South Africa, etc., (Figure 2A, Supplementary Table 1), which also showed a severe burden in ASDR (Figure 2B, Supplementary Table 1). During the period from 1990 to 2019, 24 countries and territories showed an upward trend in ASPR, including China and the USA. ASDRs in most countries and territories showed a downward trend. Only a very small number of countries and territories, such as Niger and Burkina Faso, show an upward trend both in ASPR and ASDR (Figure 2C, Figure 2D, Supplementary Table 1, Supplementary Table 2). The number of DALYs and prevalent cases was high in high-middle and middle socio-demographic index (SDI) regions and increased in all SDI regions compared with 1990. ASPR and ASDR in other SDI regions showed a downward or stable trend, excluding the rising trend of ASPR in high and high-middle SDI regions (Table 1, Supplementary Figure 1). Except for the ASPR in East Asia, which showed an upward trend, the ASPR and ASDR in other GBD regions showed a downward or stable trend (Supplementary Table 2).

### ARHL burden due to severity

Compared with 1990, the number of DALYs and prevalent cases of ARHL of all severity in 2019 has increased significantly. Among them, the number of DALYs and prevalent cases of mild ARHL was the highest, 10.28 (95% UI: 4.57, 19.82) million and 1083.06 (95% UI: 1029.88, 1136.29) million respectively, which almost doubled from 1990. The ASPR and ASDR of all other severity ARHL were lower than 0.1/1000 and 5/100, and both showed a downward trend, except for mild ARHL. The ASDR and ASPR of mild ARHL were 0.12/1000 and 13.09/100 in 2019, respectively, and both showed an upward trend, with EAPC of 0.18 and 0.18 respectively (Table 1). In terms of age distribution, people over 75 years old account for a larger proportion of moderate/moderately severe/severe ARHL, and people under 49 years old account for a larger proportion of profound/complete ARHL (Supplementary Figure 2). Except for the DALY rate of mild ARHL and the moderately severe ARHL of the age group over 70 years old, which showed a slight increase, the DALY rate of ARHL of each severity of all age groups showed a downward trend or remained unchanged (Supplementary Figure 3A). Except for the increase in DALY rate attributable to occupational noise for males aged 15–49 in the high/high middle SDI region, the DALY rate attributable to occupational noise of males in all age groups and SDI regions was decreasing. The DALY rate attributable to occupational noise for females aged 15–49 in all SDI regions and females over 50 in high/high-middle SDI regions was increasing (Supplementary Figure 3B).

### The influential factors for EAPC

**Figure 3:**
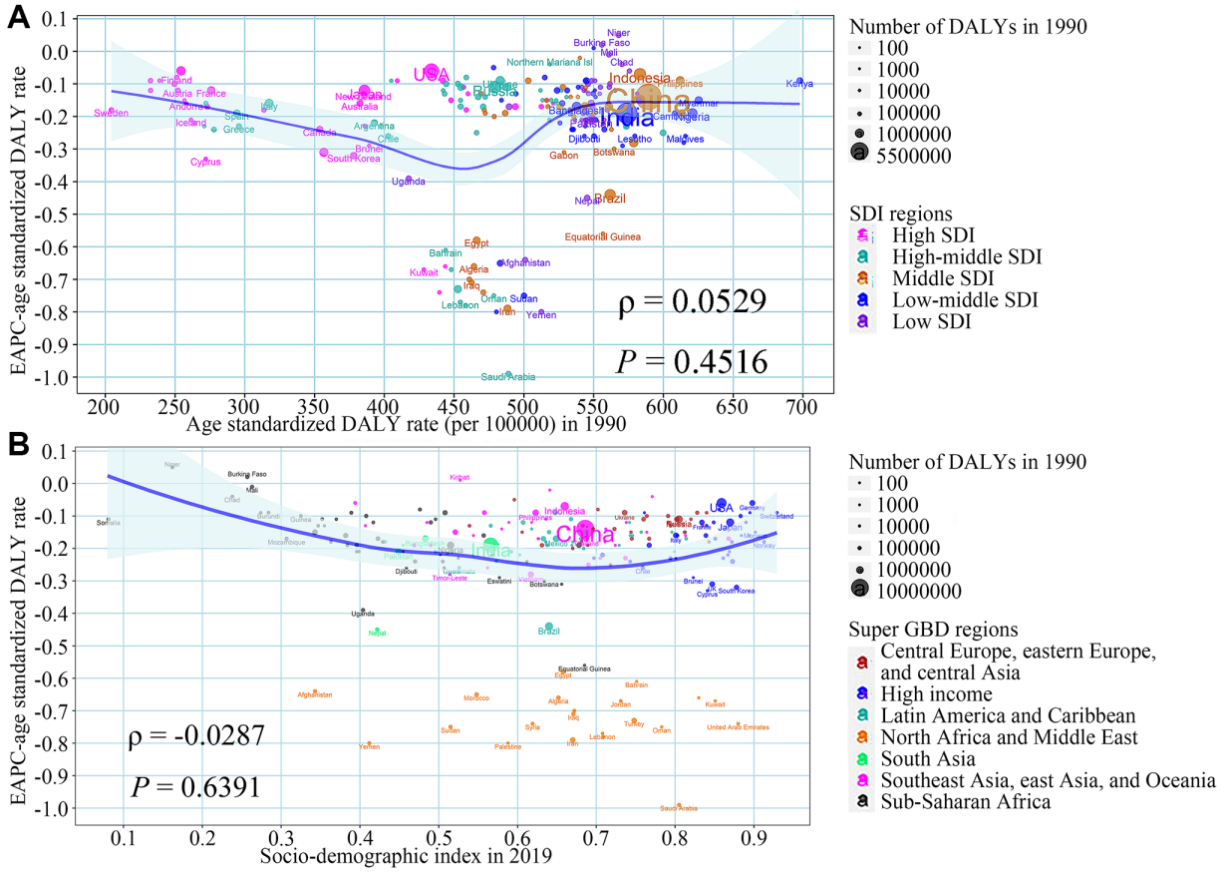
**The association between ASDR in 1990, SDI in 2019, and the EAPC of ASDR from 1990 to 2019.** (**A**) ASDR in 1990 and the EAPC of ASDR from 1990 to 2019; (**B**) SDI in 2019 and the EAPC of ASDR from 1990 to 2019. The blue line was an adaptive association fitted with adaptive Loess regression based on all data points. Abbreviations: EAPC: estimated annual percentage change; SDI: socio-demographic index; ASDR: age-standardized DALY rate; DALY: disability-adjusted life year.

Figure 3 shows the impact of the ASDR in 1990 and SDI in 2019 on ASDR trends in each region. There was no significant correlation between ASDR of ARHL in 1990 and EAPC of ASDR from 1990 to 2019 at the national level (ρ = 0.0529, *P* = 0.4516), indicating that ARHL burden may not be given priority intervention in countries with high burden rates. Compared with other countries, some countries with a medium disease burden, such as Egypt, Iran, had a faster decline in disease burden (Figure 3A). Similarly, the temporal trend of ARHL related ASDR from 1990 to 2019 and SDI in 2019 still seemed to have no significant associations at the national level (ρ = −0.0287, *P* = 0.6391). Some countries in North Africa and Middle East had the most obvious downward trend, despite their varying SDI in 2019 (Figure 3B). In terms of ASPR, we found a positive correlation between the ASPR in 1990 and the EAPC of ASPR from 1990 to 2019 at the national level (ρ = 0.2437, *P* = 0.0004) (Supplementary Figure 4). Further, we investigated the correlation between SDI and ASDR by severity from 1990 to 2019 in 21 GBD regions around the world. The results showed that, except for the burden of mild ARHL, which showed a significant upward trend with the increase of SDI, the ASDR of ARHL in other severity was significantly negatively correlated with SDI in the 21 GBD regions (Supplementary Figure 5).

### The influential factors for ARHL burden attributable to occupational noise

**Figure 4:**
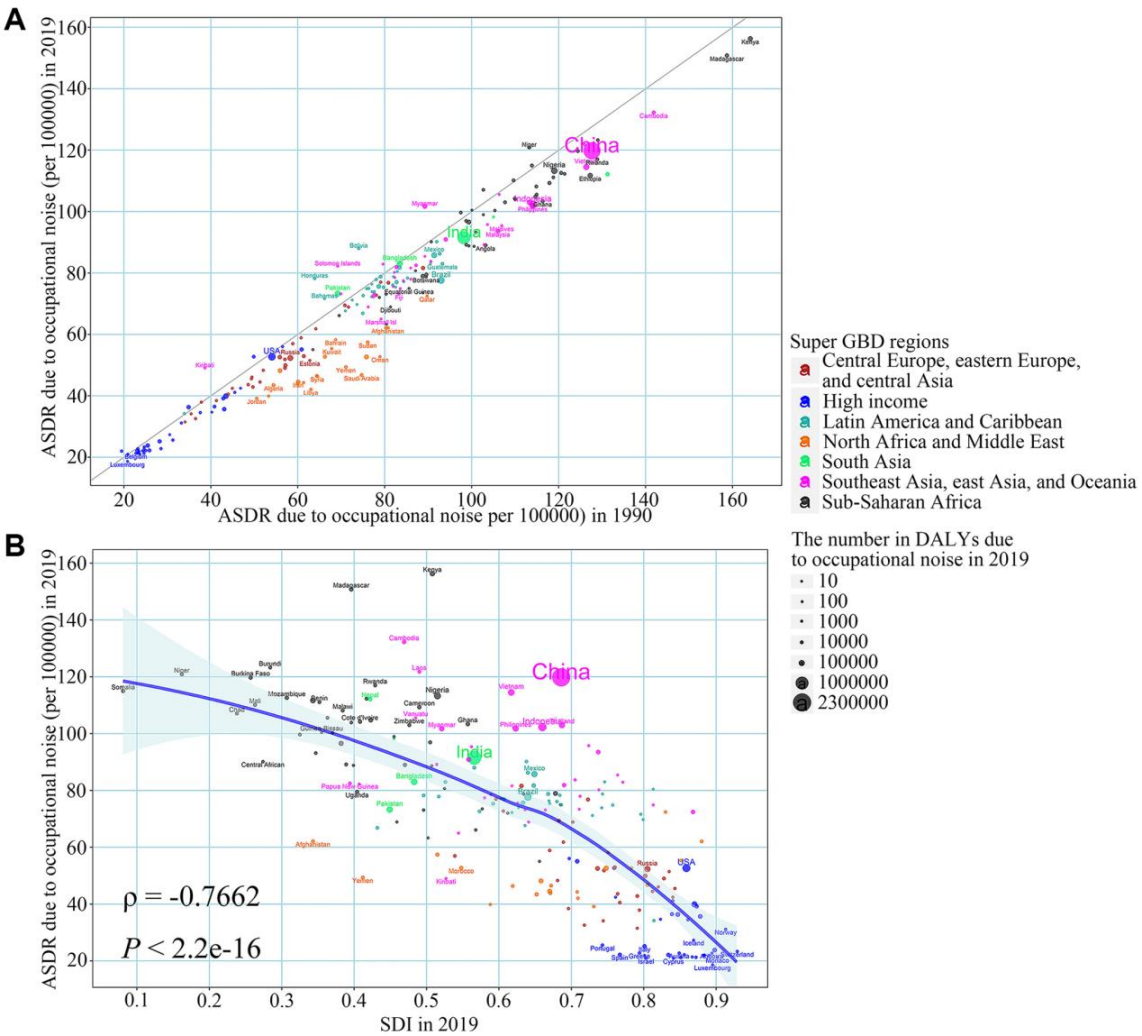
**The relationship between ASDR due to occupational noise in 1990, SDI in 2019 and ASDR due to occupational noise in 2019 in age-related hearing loss.** (**A**) ASDR due to occupational noise in 1990; (**B**) SDI in 2019. The blue line was an adaptive association fitted with adaptive Loess regression based on all data points. Abbreviations: ASDR: age-standardized DALY rates; SDI: socio-demographic index; DALY: disability-adjusted life year.

Among 204 countries and territories, the ASDR attributable to occupational noise in most countries in 2019 was lower than that in 1990 and countries with increased disease burden due to occupational noise were mostly in East Asia and Latin America and Caribbean (Figure 4A). With the increase in SDI in 2019, the ASDR attributed to occupational noise in 2019 was declining at the national level (ρ = −0.7662, *P* < 2.2e-16), which reflected the countries with higher SDI levels may be better able to reduce the burden of ARHL (Figure 4B). Similar results were observed in the aspects of the proportion of ARHL burden attributable to occupational noise (Supplementary Figure 6).

## DISCUSSION

Our study summarized the ARHL disease burden by gender, age groups, and severity at the global, regional, and national levels, and further discuss the temporal trends and the influencing factors over the past three decades. Overall, the number of ARHL prevalent cases and DALYs in the world nearly doubled from 1990 to 2019. The ASPR of 24 countries and territories is on the rise, and some of them have large populations, such as the USA and China. The ASDR of most countries and territories shows a downward trend. Mild ARHL accounted for the largest proportion of all ARHL, and only mild ARHL showed an upward trend in ASDR and ASPR. The proportion of ASDR attributed to occupational noise was on the decline in most regions, and with the increase of SDI, the proportion of ASDR attributed to occupational noise was on the decline.

In the past 30 years, the disease burden of ARHL has almost doubled. In 2019, the number of ARHL cases worldwide reached 1456.66 million, which is equivalent to almost one in every five people suffering from ARHL. From 1990 to 2019, the number of people over the age of 30 in the world increased from 2157.4 million to 3825.4 million, almost doubling, however, the number of people under the age of 30 changed slightly (from 3237.4 million to 3815.1 million) [6]. The aging population and the higher prevalence rate of ARHL among people over 45 years old may be the reasons for the nearly doubled prevalent cases of ARHL. Previous studies have shown that the disease burden of hearing loss in low SDI countries is heavier than that in high SDI countries, and our research supports this view [5]. Complete occupational noise control regulations, a sufficient number of otolaryngologists, adequate financial support, and accurate disease burden data in high SDI countries can all help these countries reduce the burden of ARHL. In addition, the improvement of the global economy has increased people’s purchasing power [7], making some people more willing to spend money on medical services that were unaffordable in the past, such as hearing aids and cochlear implants [8]. This not only improves the hearing level of these people, but also reduces the disease burden of ARHL in these countries where the economy is improving. However, the huge economic impact worldwide brought about by the COVID-19 pandemic and the almost irreversible population aging trend will bring severe challenges to the prevention and control of ARHL. Countries around the world need to work together to take a series of measures to reduce the burden of disease caused by ARHL.

Our study found that mild hearing loss accounted for a large proportion of all ARHL patients, and the ASPR and ASDR of mild ARHL showed an upward trend. The ASPR and ASDR of other severity ARHL showed a downward trend. In recent years, some scholars began to call for hearing screening for the elderly [9]. In 2012, U.S. Preventive Services Task Force published a guideline on hearing screening for people over the age of 50, which detailed the risk assessment, screening test, interventions, and balance of harms and benefits [10]. The progress of hearing screening provided opportunities for the discovery of many patients with mild ARHL. The economic situation will affect people’s behavior of seeking help from doctors [11]. The improvement of economic conditions makes people no longer suffer the pain of hearing loss silently as in the past, and seek help from professional doctors, which also increases the chances of discovering the mild hearing loss. In addition, early detection and early treatment of ARHL may prevent a considerable number of people from developing more severe hearing loss, which also increases the number of people with mild hearing loss.

The gratifying result we found is that the ASPR and ASDR of ARHL showed a downward trend in most countries and regions. Noise exposure, especially occupational noise exposure, is one of the important risk factors of ARHL [12]. Since the Second World War in 1945, in order to reduce the impact of occupational noise on hearing loss, humans have adopted measures such as the use of protective equipment, the formulation of regulations and supervision [13]. Obvious downtrends were observed in OSHA occupational noise exposure measurements from 1979 to 2013 [14]. In our study, the burden of ARHL attributable to occupational noise in most countries has shown a downward trend, which also supports the view that noise levels have fallen to a certain extent. Further, some countries, such as Colombia, Brazil, and Chile, have public programs that can provide hearing aids for the elderly, which can further reduce the disease burden of ARHL [8]. In addition, past studies have shown that malnutrition is a potential risk factor for ARHL [15]. The growing economy in some regions may improve the diet of local residents, thereby reducing the incidence of ARHL [16–18]. We also found that countries and territories with higher SDI had a lower disease burden of ARHL attributable to occupational noise. Compared with low SDI countries, high SDI countries have better noise regulations and wider use of protective gear. These may be the reasons for the lower ARHL disease burden attributed to noise in high-SDI countries.

This study has some limitations. First, although GBD collaborators have done a lot of work in assessing the annual burden of disease around the world, they cannot avoid bias in modeling the unavailable data [19, 20]. Second, due to the limitation of the database, our data may contain a very small amount of hearing loss data caused by other reasons, such as the use of ototoxic drugs or trauma [3, 21], which only have little influence on the overall analysis.

In summary, in the past 30 years, ASRs of ARHL in most countries and regions have shown a downward trend, with mild ARHL accounting for most of all ARHL. The ARHL disease burden showed a downward trend with the increase of SDI, and the burden of ARHL due to occupational noise also showed a downward trend. However, as the population ages intensified, the absolute disease burden of ARHL is still heavy. Countries around the world should take effective measures to reduce the ARHL disease burden.

## METHODS

Previous studies have reported the details of the GBD study [1, 19, 20], and the methods we present here are specific to the ARHL disease burden estimation. Guidelines for Accurate and Transparent Health Estimates Reporting (GATHER) guidelines were followed in every step of analyzing the GBD database [22].

### Study data

In our study, we collected annual prevalent cases, ASPR, DALYs and ASDR of ARHL by 5-year age groups, genders, severity, regions, and risk factors from 1990 to 2019 from the GBD 2019 database via the Global Health Data Exchange (GHDx) query tool (http://ghdx.healthdata.org/gbd-results-tool). Hearing loss was defined as the average intensity of the softest sound that can be heard in one or both ears at a frequency of 0.5, 1, 2, and 4 kHz equal to or greater than 20 dB. According to the average intensity, hearing loss can be divided into mild (threshold in 20–34 dB), moderate (threshold in 35–49 dB), moderately severe (threshold in 50–64 dB), severe (threshold in 65–79 dB), profound (threshold in 80–94 dB) and complete (threshold more than 95 dB). The data used in this study excluded hearing loss caused by congenital, meningitis, and otitis media. The occupational noise exposure was defined as the proportion of the population occupationally exposed to 85+ decibels of noise based on population distributions across 17 economic activities, such as agriculture, hunting, forestry, fishing, mining and quarrying. The burden of ARHL for occupational noise was estimated using the population attributable fraction (PAF), which requires information on the relative risk of hearing loss due to occupational noise and occupational noise exposure levels. The relative risks were obtained from published meta-analyses or pooled studies and the formula of PAF was:


$$PAF=\frac{\sum_{x=1}^{n}RR(x)P(x)-1}{\sum_{x=1}^{n}RR(x)P(x)}$$

where *P(x)* is the proportion of population exposed to occupational noise at level x in the target population and *RR(x)* is the relative risk of occupational noise exposure level x [23, 24]. In the GBD database, a total of 204 countries and territories were included and were classified into 5 regions according to their SDIs, which were calculated by combining educational attainment, total fertility rate, and the lag-distributed income per capita. Further, the 204 countries and territories were divided into 21 GBD regions based on geographical proximity and epidemiological similarity, and further simplified into seven Super GBD regions.

Previous studies have introduced the method of disease burden estimation detailly. In short, DisMod MR 2.1 was used to estimate the ARHL disease burden based on published literature and cross-sectional studies. The exposure to risk factors and its attributable burden were quantified by GBD comparative risk assessment [1, 19, 20]. The age-standardized rates (ASRs) were estimated according to the world population by the GBD study. Relevant data were reported in numbers and 95% UIs, which were determined by 2.5% and 97.5% of the ordered 1000 estimates.

### Statistical analysis

We applied ASPR, ASDR, and EAPC to quantify the trends of ARHL disease burden by age, gender, regions, severity, and risk factors from 1990 to 2019. Standardization is important for this study because it can avoid the difference in age compositions of different groups even of the same population in different periods. The EAPC, a widely used measure that summarized the ASR trends in a specified time interval, was calculated to describe the temporal trends of ASRs of ARHL burden. We put ASR in the regression line model “ln (ASR) = α + β × calendar year + ɛ”. The calculation formula of EAPC is 100 × (exp (β)-1). The 95% CI for EAPC is also generated from this model. If both the EAPC estimate and the lower boundary of its 95% CI are greater than 0, the ASR is considered to be on the rise. Conversely, if the upper boundary of the EAPC estimate and its 95% CI is less than 0, then ASR is considered to be a downward trend. Otherwise, ASR is considered stable over time [25–27]. In order to explore the influencing factors of EAPC, we used Spearman rank test to evaluate the relationship between EAPC and ASR (1990) and SDI (2019) respectively at the national level. ASR (1990) reflects the initial disease burden, and SDI (2019) can replace the socioeconomic level and availability of medical services in different countries/territories [25–27]. R program was used in all statistical analyses of our study (version 4.0.3; https://www.R-project.org/), and the *P*-value on both sides of less than 0.05 was considered statistically significant.

### Data sharing statement

To download the data used in these analyses, please visit the Global Health Data Exchange GBD 2019 website: http://ghdx.healthdata.org/gbd-results-tool.

### Ethical statement

Our study protocol was approved by the Institutional Review Boards of Qilu Hospital of Shandong University with approval number KYLL-202011(KS)-239.

## Supplementary Materials

Supplementary Figures

Supplementary Table 1

Supplementary Table 2
