# Supplementary material for: Global, regional, and national burden of age-related hearing loss from 1990 to 2019
Source: Aging (Albany NY). 2021 Dec 15;13(24):25944–59. doi: 10.18632/aging.203782 (PMC8751586; doi:10.18632/aging.203782)
Supplement: Supplementary Figures [file aging-13-203782-s001.pdf]

SUPPLEMENTARY FIGURES

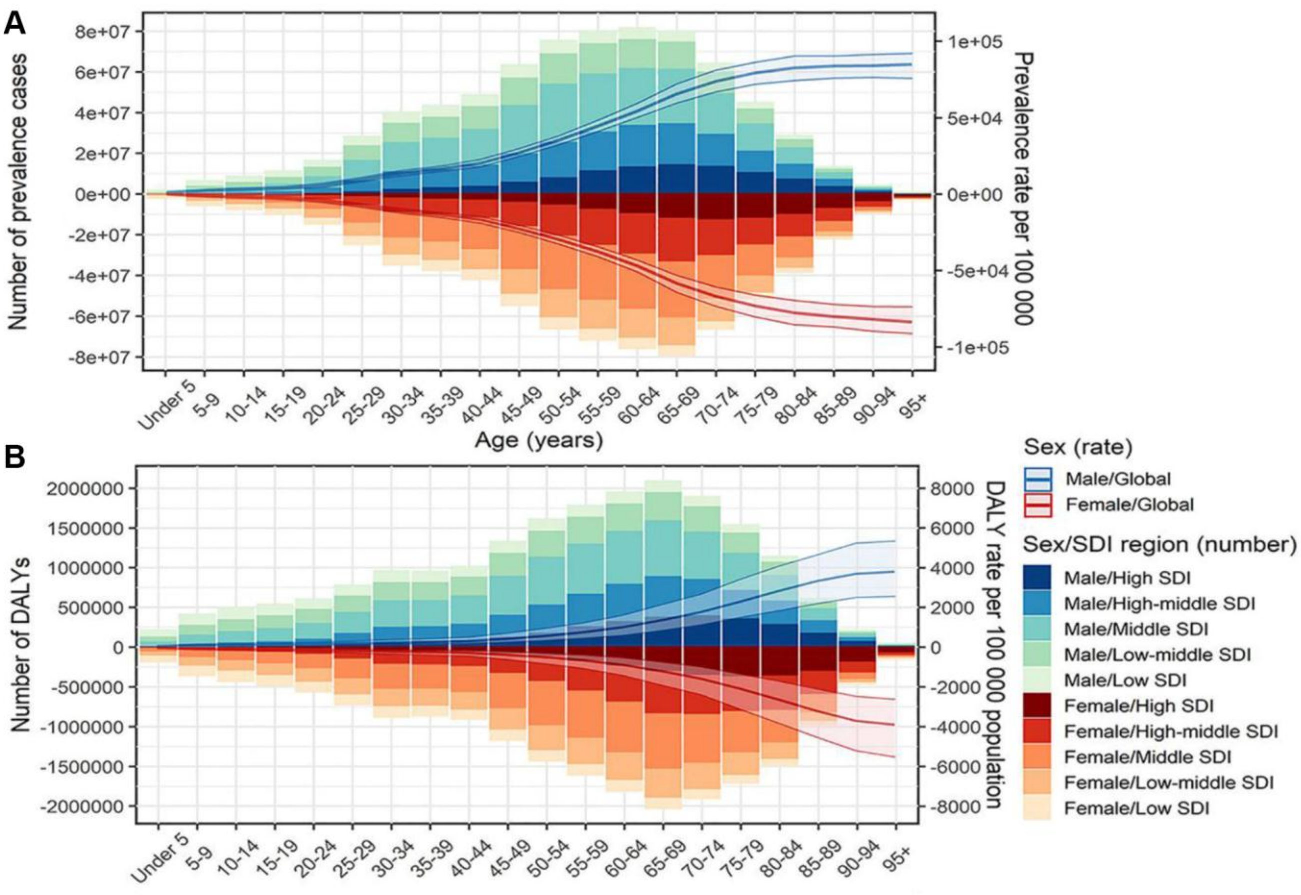

Supplementary Figure 1. Prevalent cases, DALYs and the corresponding rates of ARHL by sex, age group, and SDI regions in 2019. (**A**) Prevalent cases and prevalence rate; (**B**) DALYs and DALY rate. Abbreviations: SDI: socio-demographic index; ARHL: age-related hearing loss; DALY: disability adjusted life year.

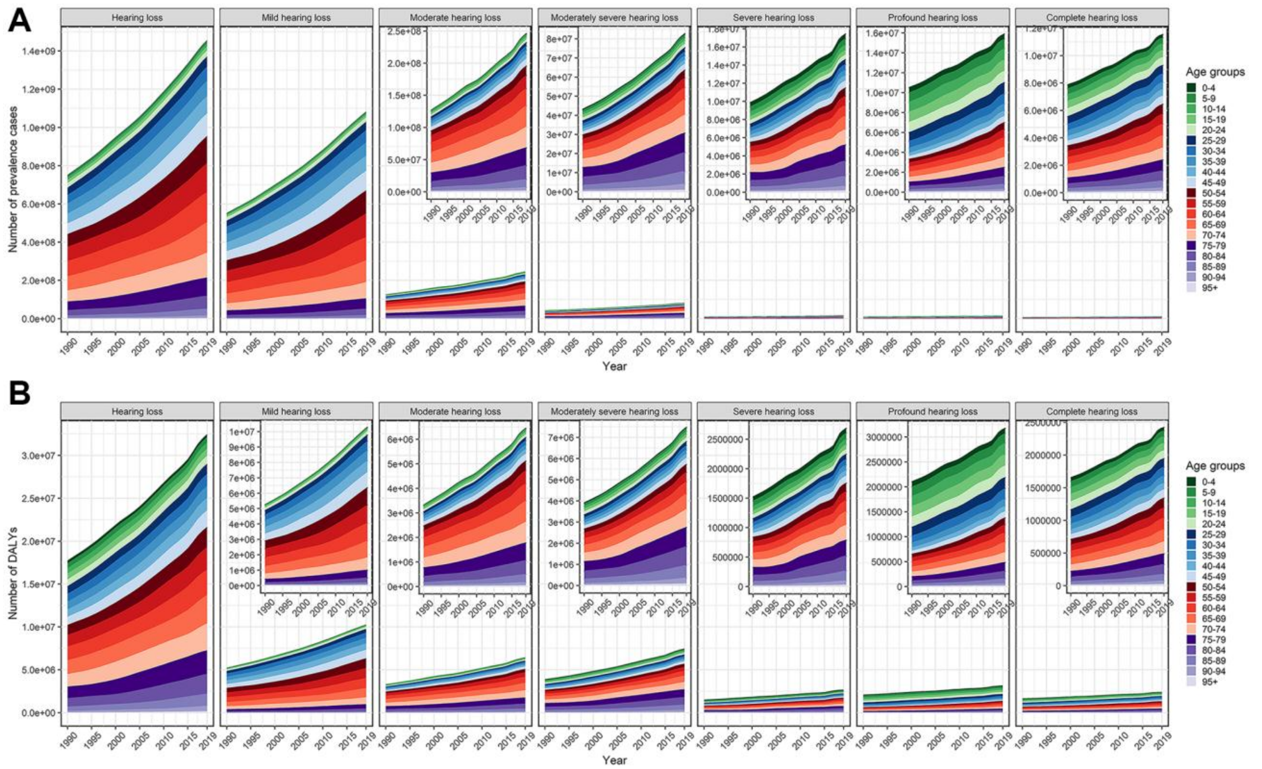

Supplementary Figure 2. The prevalent cases and DALYs of ARHL by severity and age groups from 1990 to 2019. (**A**) Prevalent cases; (**B**) DALYs. Abbreviations: ARHL: age-related hearing loss; DALY: disability adjusted life year.

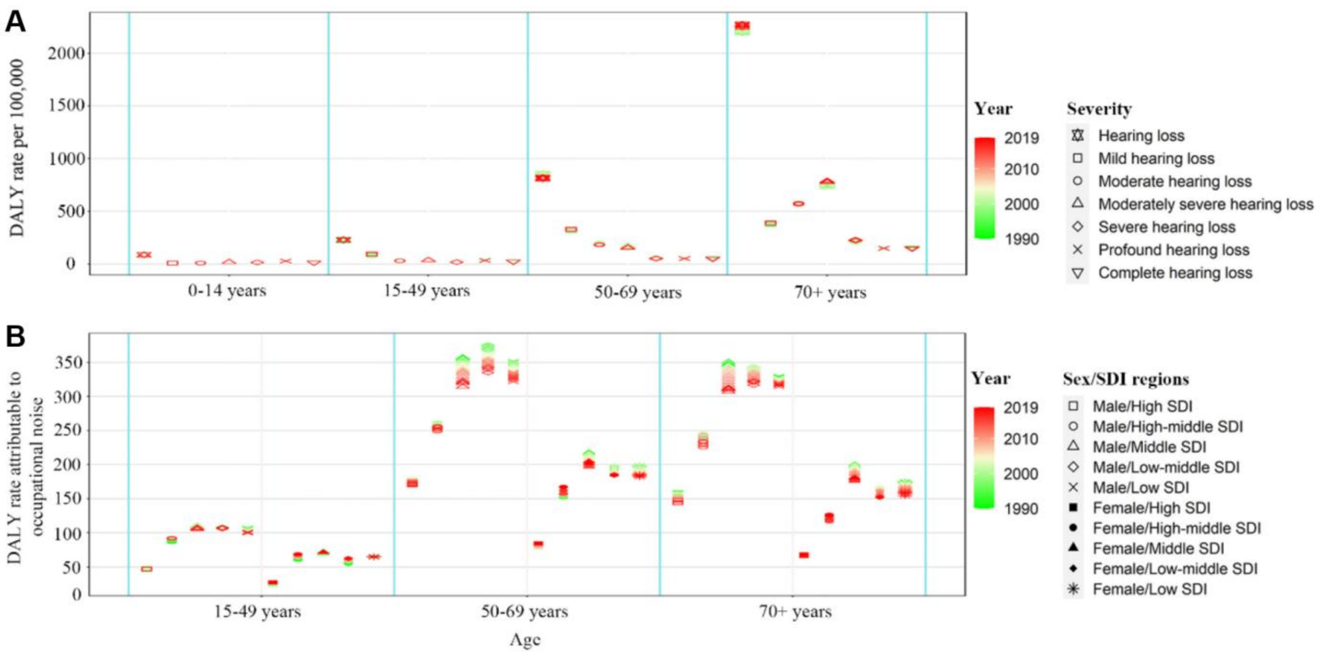

Supplementary Figure 3. The trends of DALY rate and DALY rate attributable to occupational noise by sex, age groups, severity, and SDI regions from 1990 to 2019. (**A**) DALY rate by age groups and severity; (**B**) DALY rate attributable to occupational noise by sex, age groups and SDI regions. Abbreviations: SDI: socio-demographic index; DALY: disability adjusted life year.

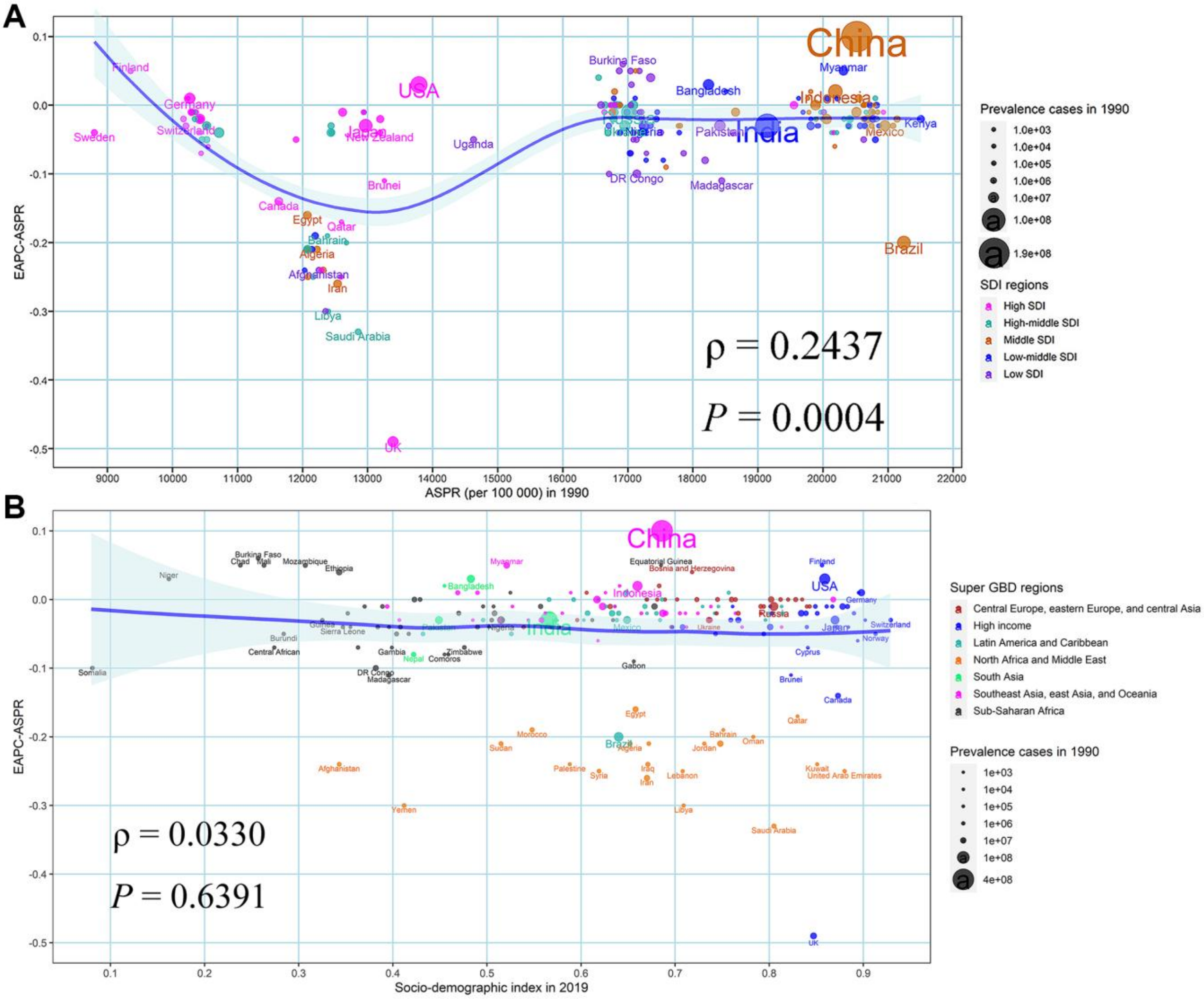

Supplementary Figure 4. The association between ASPR in 1990, SDI in 2019, and the EAPC of ASPR from 1990 to 2019. (**A**) ASPR in 1990 and the EAPC of ASPR from 1990 to 2019; (**B**) SDI in 2019 and the EAPC of ASPR from 1990 to 2019. The blue line was an adaptive association fitted with adaptive Loess regression based on all data points. Abbreviations: EAPC: estimated annual percentage change; ASPR: age-standardized prevalence rate; SDI: socio-demographic index.

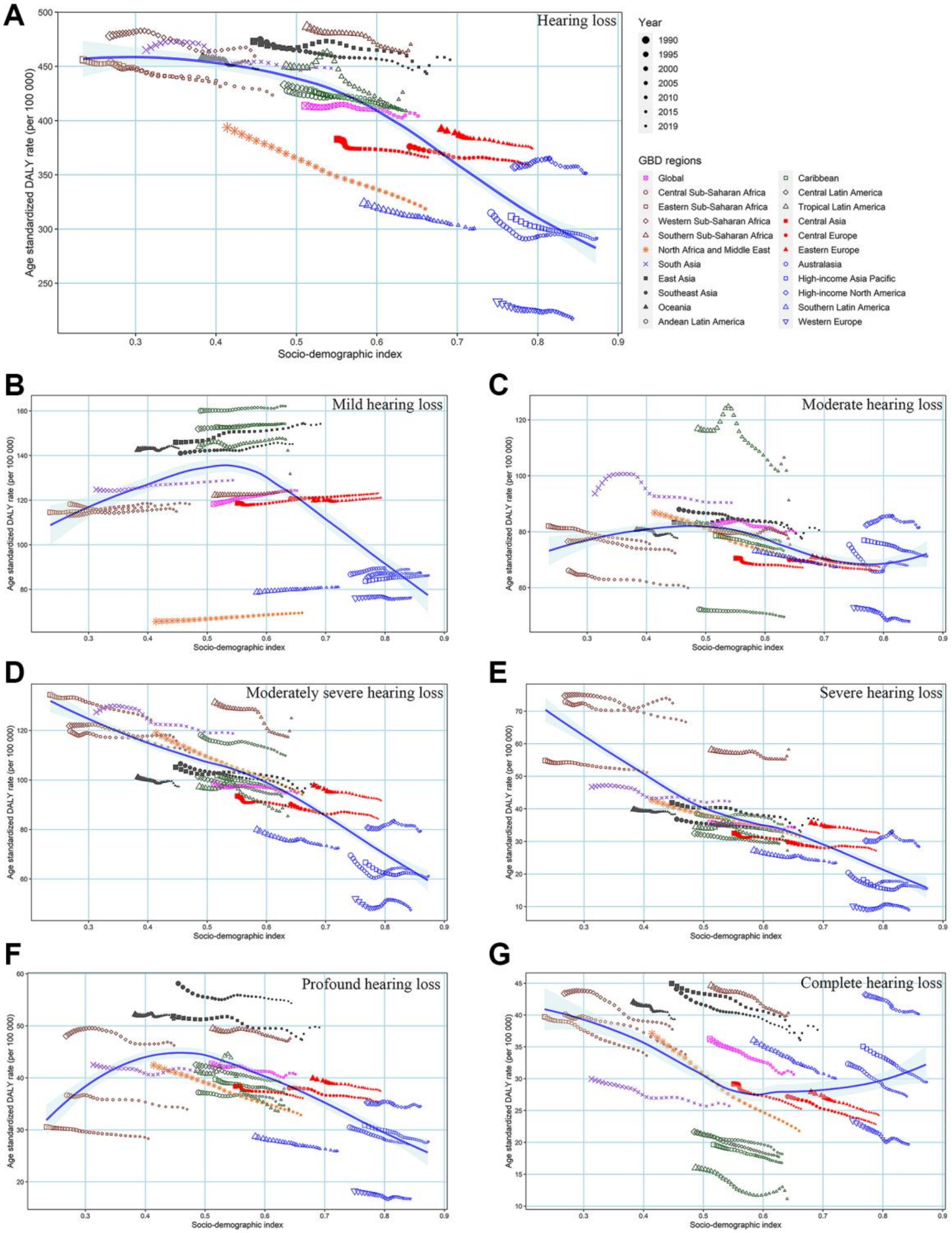

Supplementary Figure 5. The association between ASDR and SDI by GBD regions and severity from 1990 to 2019. (**A**) total ARHL; (**B**) mild ARHL; (**C**) moderate ARHL; (**D**) moderately severe ARHL; (**E**) severe ARHL; (**F**) profound ARHL; (**G**) complete ARHL. The blue line was an adaptive association fitted with adaptive Loess regression based on all data points. Abbreviations: ASDR: age-standardized DALY rates; SDI: socio-demographic index; DALY: disability adjusted life year; ARHL: age-related hearing loss; GBD: global burden of disease.

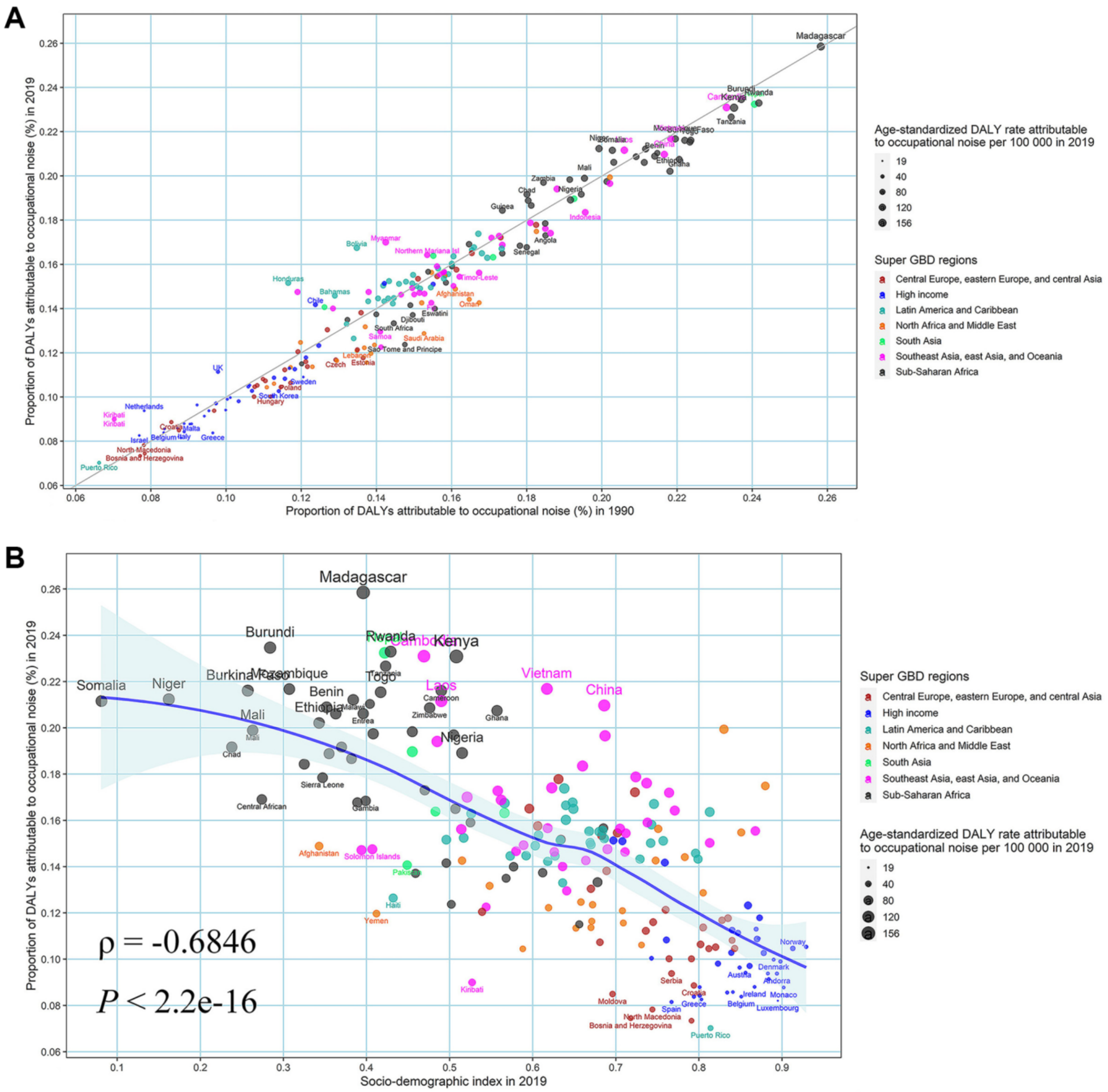

Supplementary Figure 6. The relationship between proportion of DALYs attributable to occupational noise in 1990, SDI in 2019 and proportion of DALYs attributable to occupational noise in 2019 in ARHL. (**A**) Proportion of DALYs attributable to occupational noise in 1990; (**B**) SDI in 2019. The blue line was an adaptive association fitted with adaptive Loess regression based on all data points. Abbreviations: DALY: disability adjusted life year; SDI: socio-demographic index; ARHL: age-related hearing loss.
