# Supplementary material for: Global, regional, and national burden of age-related hearing loss from 1990 to 2019
Source: Aging (Albany NY). 2021 Dec 15;13(24):25944–59. doi: 10.18632/aging.203782 (PMC8751586; doi:10.18632/aging.203782)
Supplement: Supplementary Table 1 [file aging-13-203782-s002.docx]

**Supplementary Table 1. Age-related hearing loss prevalent cases and burden in 1990 and 2019 and the temporal trends from 1990 to 2019 in 204 countries.**

| **Characteristics** | **1990** | | | | |  | **2019** | | | | |  | **EAPC (1990–2019)** | | |  |
| --- | --- | --- | --- | --- | --- | --- | --- | --- | --- | --- | --- | --- | --- | --- | --- | --- |
|  | **Prevalent cases** | |  | **DALYs** | |  | **Prevalent cases** | |  | **DALYs** | |  | **ASPR** |  | **ASDR** | |
|  |  | **ASPR** |  |  | **ASDR** |  |  | **ASPR** |  |  | **ASDR** |  |  |  |  | |
|  | **No.×10^6^**  **(95% UI)** | **No.×10^-2^**  **(95% UI)** |  | **No.×10^6^**  **(95% UI)** | **No.×10^-3^**  **(95% UI)** |  | **No.×10^6^**  **(95% UI)** | **No.×10^-2^**  **(95% UI)** |  | **No.×10^6^**  **(95% UI)** | **No.×10^-3^**  **(95% UI)** |  | **No.**  **(95% CI)** |  | **No.**  **(95% CI)** | |
| Afghanistan | 0.90  (0.84, 0.96) | 12.25  (11.52, 12.98) |  | 0.04  (0.03, 0.05) | 5.01  (3.53, 6.89) |  | 1.83  (1.66, 1.99) | 11.36  (10.66, 12.04) |  | 0.07  (0.05, 0.10) | 4.17  (2.99, 5.72) |  | -0.24  (-0.27, -0.21) |  | -0.64  (-0.66, -0.62) | |
| Albania | 0.40  (0.38, 0.43) | 16.75  (15.94, 17.58) |  | 0.01  (0.01, 0.02) | 4.72  (3.17, 6.73) |  | 0.64  (0.61, 0.67) | 16.75  (16.00, 17.54) |  | 0.02  (0.01, 0.02) | 4.50  (3.06, 6.43) |  | 0.00  (0.00, 0.01) |  | -0.18  (-0.19, -0.16) | |
| Algeria | 1.72  (1.61, 1.83) | 12.22  (11.50, 12.92) |  | 0.07  (0.05, 0.09) | 4.64  (3.31, 6.41) |  | 4.13  (3.90, 4.38) | 11.48  (10.87, 12.15) |  | 0.13  (0.09, 0.19) | 3.83  (2.67, 5.37) |  | -0.21  (-0.22, -0.20) |  | -0.66  (-0.68, -0.63) | |
| American Samoa | 0.01  (0.01, 0.01) | 20.13  (19.39, 20.90) |  | 0.00  (0.00, 0.00) | 5.31  (3.60, 7.53) |  | 0.01  (0.01, 0.01) | 19.80  (19.09, 20.52) |  | 0.00  (0.00, 0.00) | 5.05  (3.41, 7.19) |  | -0.04  (-0.06, -0.03) |  | -0.14  (-0.15, -0.12) | |
| Andorra | 0.01  (0.01, 0.01) | 10.55  (9.96, 11.14) |  | 0.00  (0.00, 0.00) | 2.58  (1.71, 3.77) |  | 0.01  (0.01, 0.01) | 10.39  (9.83, 10.98) |  | 0.00  (0.00, 0.00) | 2.43  (1.60, 3.61) |  | -0.06  (-0.06, -0.05) |  | -0.16  (-0.18, -0.13) | |
| Angola | 0.99  (0.93, 1.05) | 17.44  (16.63, 18.22) |  | 0.03  (0.02, 0.05) | 5.58  (3.84, 7.86) |  | 2.88  (2.71, 3.05) | 17.15  (16.41, 17.90) |  | 0.09  (0.06, 0.13) | 5.14  (3.55, 7.26) |  | -0.02  (-0.04, -0.01) |  | -0.24  (-0.26, -0.22) | |
| Antigua and Barbuda | 0.01  (0.01, 0.01) | 20.82  (19.67, 22.03) |  | 0.00  (0.00, 0.00) | 5.25  (3.49, 7.63) |  | 0.02  (0.02, 0.02) | 20.71  (19.56, 21.93) |  | 0.00  (0.00, 0.00) | 4.98  (3.31, 7.27) |  | -0.02  (-0.02, -0.01) |  | -0.16  (-0.17, -0.15) | |
| Argentina | 3.99  (3.78, 4.21) | 12.44  (11.78, 13.13) |  | 0.12  (0.09, 0.18) | 3.93  (2.71, 5.56) |  | 6.40  (6.06, 6.75) | 12.29  (11.64, 12.93) |  | 0.19  (0.13, 0.27) | 3.65  (2.50, 5.19) |  | -0.04  (-0.04, -0.03) |  | -0.22  (-0.24, -0.21) | |
| Armenia | 0.49  (0.47, 0.51) | 16.74  (15.97, 17.50) |  | 0.01  (0.01, 0.02) | 4.72  (3.20, 6.71) |  | 0.65  (0.62, 0.68) | 16.66  (15.94, 17.47) |  | 0.02  (0.01, 0.02) | 4.48  (3.03, 6.42) |  | 0.00  (-0.01, 0.02) |  | -0.19  (-0.20, -0.18) | |
| Australia | 2.53  (2.44, 2.61) | 13.19  (12.74, 13.64) |  | 0.07  (0.05, 0.10) | 3.82  (2.66, 5.43) |  | 4.84  (4.55, 5.14) | 13.00  (12.31, 13.78) |  | 0.14  (0.09, 0.19) | 3.55  (2.40, 5.08) |  | -0.02  (-0.05, 0.00) |  | -0.16  (-0.24, -0.08) | |
| Austria | 1.11  (1.04, 1.18) | 10.31  (9.73, 10.88) |  | 0.03  (0.02, 0.04) | 2.52  (1.68, 3.73) |  | 1.56  (1.47, 1.65) | 10.28  (9.72, 10.84) |  | 0.04  (0.03, 0.06) | 2.36  (1.57, 3.51) |  | -0.01  (-0.02, 0.00) |  | -0.12  (-0.16, -0.07) | |
| Azerbaijan | 0.94  (0.89, 0.99) | 16.79  (16.05, 17.58) |  | 0.03  (0.02, 0.04) | 4.69  (3.15, 6.75) |  | 1.73  (1.65, 1.82) | 16.80  (16.06, 17.57) |  | 0.04  (0.03, 0.06) | 4.53  (3.05, 6.47) |  | 0.02  (0.00, 0.04)* |  | -0.13  (-0.15, -0.12) | |
| Bahamas | 0.04  (0.04, 0.04) | 20.87  (19.76, 22.10) |  | 0.00  (0.00, 0.00) | 5.13  (3.41, 7.54) |  | 0.08  (0.08, 0.09) | 20.68  (19.53, 21.89) |  | 0.00  (0.00, 0.00) | 4.92  (3.22, 7.23) |  | -0.03  (-0.03, -0.03) |  | -0.15  (-0.16, -0.14) | |
| Bahrain | 0.03  (0.03, 0.04) | 12.38  (11.73, 13.07) |  | 0.00  (0.00, 0.00) | 4.44  (3.13, 6.16) |  | 0.16  (0.15, 0.17) | 11.76  (11.15, 12.41) |  | 0.00  (0.00, 0.01) | 3.73  (2.60, 5.21) |  | -0.19  (-0.20, -0.18) |  | -0.61  (-0.64, -0.58) | |
| Bangladesh | 10.83  (10.14, 11.53) | 18.24  (17.39, 19.15) |  | 0.34  (0.23, 0.48) | 5.38  (3.67, 7.64) |  | 25.95  (24.77, 27.26) | 18.27  (17.47, 19.14) |  | 0.71  (0.48, 1.01) | 5.07  (3.45, 7.19) |  | 0.03  (0.02, 0.04)* |  | -0.17  (-0.19, -0.15) | |
| Barbados | 0.06  (0.05, 0.06) | 20.71  (19.57, 21.95) |  | 0.00  (0.00, 0.00) | 5.12  (3.43, 7.46) |  | 0.09  (0.08, 0.10) | 20.58  (19.42, 21.81) |  | 0.00  (0.00, 0.00) | 4.92  (3.25, 7.24) |  | -0.02  (-0.02, -0.02) |  | -0.12  (-0.13, -0.12) | |
| Belarus | 2.06  (1.97, 2.16) | 16.65  (15.88, 17.46) |  | 0.06  (0.04, 0.08) | 4.71  (3.18, 6.68) |  | 2.35  (2.24, 2.47) | 16.60  (15.87, 17.41) |  | 0.06  (0.04, 0.09) | 4.47  (3.01, 6.41) |  | 0.00  (-0.01, 0.01) |  | -0.17  (-0.18, -0.16) | |
| Belgium | 1.46  (1.38, 1.55) | 10.42  (9.88, 11.01) |  | 0.04  (0.03, 0.06) | 2.73  (1.84, 3.99) |  | 1.99  (1.88, 2.10) | 10.34  (9.81, 10.88) |  | 0.05  (0.04, 0.08) | 2.54  (1.70, 3.70) |  | -0.02  (-0.03, -0.02) |  | -0.17  (-0.20, -0.13) | |
| Belize | 0.02  (0.02, 0.02) | 20.85  (19.68, 22.15) |  | 0.00  (0.00, 0.00) | 5.57  (3.71, 8.07) |  | 0.07  (0.06, 0.07) | 20.66  (19.49, 21.92) |  | 0.00  (0.00, 0.00) | 5.18  (3.46, 7.49) |  | -0.03  (-0.03, -0.03) |  | -0.22  (-0.24, -0.21) | |
| Benin | 0.46  (0.43, 0.50) | 17.21  (16.43, 18.04) |  | 0.02  (0.01, 0.02) | 5.55  (3.82, 7.78) |  | 1.22  (1.13, 1.30) | 17.00  (16.24, 17.79) |  | 0.04  (0.03, 0.06) | 5.32  (3.61, 7.51) |  | -0.02  (-0.04, 0.00) |  | -0.11  (-0.13, -0.10) | |
| Bermuda | 0.01  (0.01, 0.01) | 20.93  (19.80, 22.16) |  | 0.00  (0.00, 0.00) | 5.17  (3.45, 7.52) |  | 0.02  (0.02, 0.02) | 20.80  (19.62, 21.96) |  | 0.00  (0.00, 0.00) | 4.92  (3.27, 7.29) |  | -0.01  (-0.02, -0.01) |  | -0.15  (-0.16, -0.15) | |
| Bhutan | 0.06  (0.06, 0.07) | 18.51  (17.67, 19.40) |  | 0.00  (0.00, 0.00) | 5.45  (3.73, 7.75) |  | 0.12  (0.12, 0.13) | 18.65  (17.83, 19.50) |  | 0.00  (0.00, 0.00) | 5.18  (3.5, 7.37) |  | 0.02  (0.02, 0.03)* |  | -0.19  (-0.19, -0.18) | |
| Bolivia | 0.80  (0.75, 0.85) | 20.72  (19.50, 22.05) |  | 0.02  (0.01, 0.03) | 5.50  (3.75, 7.94) |  | 2.02  (1.91, 2.15) | 20.68  (19.47, 21.95) |  | 0.05  (0.03, 0.07) | 5.26  (3.57, 7.58) |  | 0.00  (-0.01, 0.00) |  | -0.13  (-0.14, -0.12) | |
| Bosnia and Herzegovina | 0.72  (0.69, 0.76) | 16.55  (15.79, 17.42) |  | 0.02  (0.01, 0.03) | 4.66  (3.17, 6.68) |  | 0.86  (0.82, 0.90) | 16.66  (15.93, 17.46) |  | 0.02  (0.02, 0.03) | 4.38  (2.97, 6.27) |  | 0.04  (0.03, 0.04)* |  | -0.25  (-0.27, -0.22) | |
| Botswana | 0.14  (0.13, 0.14) | 17.78  (17.00, 18.61) |  | 0.00  (0.00, 0.01) | 5.65  (3.88, 7.94) |  | 0.32  (0.30, 0.34) | 17.67  (16.92, 18.49) |  | 0.01  (0.01, 0.01) | 5.19  (3.58, 7.31) |  | -0.03  (-0.04, -0.03) |  | -0.30  (-0.32, -0.29) | |
| Brazil | 22.57  (21.42, 23.80) | 21.24  (20.21, 22.34) |  | 0.58  (0.39, 0.85) | 5.62  (3.75, 8.12) |  | 44.46  (42.69, 46.32) | 18.53  (17.78, 19.30) |  | 1.10  (0.72, 1.59) | 4.66  (3.07, 6.72) |  | -0.20  (-0.30, -0.11) |  | -0.44  (-0.56, -0.32) | |
| Brunei | 0.02  (0.02, 0.02) | 13.26  (12.60, 13.94) |  | 0.00  (0.00, 0.00) | 3.89  (2.65, 5.56) |  | 0.05  (0.04, 0.05) | 12.83  (12.16, 13.50) |  | 0.00  (0.00, 0.00) | 3.54  (2.41, 5.08) |  | -0.11  (-0.11, -0.10) |  | -0.29  (-0.31, -0.27) | |
| Bulgaria | 1.92  (1.83, 2.01) | 16.79  (16.09, 17.57) |  | 0.05  (0.03, 0.07) | 4.53  (3.06, 6.47) |  | 1.99  (1.89, 2.09) | 16.74  (16.00, 17.53) |  | 0.05  (0.04, 0.08) | 4.36  (2.94, 6.24) |  | 0.00  (-0.01, 0.01) |  | -0.10  (-0.12, -0.09) | |
| Burkina Faso | 0.94  (0.88, 1.00) | 16.92  (16.12, 17.77) |  | 0.03  (0.02, 0.05) | 5.56  (3.83, 7.79) |  | 2.24  (2.10, 2.40) | 17.09  (16.33, 17.92) |  | 0.08  (0.05, 0.11) | 5.54  (3.80, 7.79) |  | 0.06  (0.04, 0.08)* |  | 0.02  (0.00, 0.04)* | |
| Burundi | 0.53  (0.49, 0.57) | 17.13  (16.28, 18.00) |  | 0.02  (0.01, 0.02) | 5.44  (3.71, 7.67) |  | 1.11  (1.02, 1.19) | 16.85  (16.02, 17.69) |  | 0.04  (0.02, 0.05) | 5.25  (3.58, 7.39) |  | -0.05  (-0.05, -0.04) |  | -0.09  (-0.10 -0.08) | |
| Cambodia | 1.20  (1.14, 1.28) | 20.07  (19.25, 20.93) |  | 0.04  (0.03, 0.06) | 6.08  (4.14, 8.63) |  | 2.80  (2.68, 2.93) | 20.05  (19.27, 20.85) |  | 0.08  (0.05, 0.11) | 5.73  (3.87, 8.20) |  | 0.01  (0.00, 0.03)* |  | -0.19  (-0.20, -0.17) | |
| Cameroon | 1.04  (0.97, 1.10) | 17.19  (16.45, 17.99) |  | 0.03  (0.02, 0.05) | 5.31  (3.63, 7.56) |  | 2.93  (2.76, 3.12) | 16.87  (16.15, 17.61) |  | 0.09  (0.06, 0.13) | 5.06  (3.45, 7.13) |  | -0.04  (-0.05, -0.02) |  | -0.12  (-0.14, -0.11) | |
| Canada | 3.68  (3.50, 3.85) | 11.64  (11.11, 12.18) |  | 0.11  (0.08, 0.16) | 3.54  (2.42, 5.02) |  | 6.77  (6.52, 7.04) | 11.31  (10.85, 11.79) |  | 0.21  (0.14, 0.29) | 3.32  (2.30, 4.70) |  | -0.14  (-0.16, -0.12) |  | -0.24  (-0.26, -0.23) | |
| Cape Verde | 0.04  (0.04, 0.04) | 17.11  (16.34, 17.90) |  | 0.00  (0.00, 0.00) | 5.44  (3.68, 7.71) |  | 0.08  (0.08, 0.09) | 17.07  (16.37, 17.85) |  | 0.00  (0.00, 0.00) | 5.07  (3.46, 7.18) |  | 0.01  (-0.01, 0.03) |  | -0.23  (-0.25, -0.21) | |
| Central African Republic | 0.27  (0.25, 0.29) | 17.04  (16.24, 17.84) |  | 0.01  (0.01, 0.01) | 5.51  (3.80, 7.66) |  | 0.51  (0.47, 0.54) | 16.63  (15.78, 17.41) |  | 0.02  (0.01, 0.02) | 5.33  (3.71, 7.46) |  | -0.07  (-0.08, -0.06) |  | -0.09  (-0.11, -0.08) | |
| Chad | 0.61  (0.57, 0.65) | 17.18  (16.37, 18.02) |  | 0.02  (0.01, 0.03) | 5.70  (3.90, 8.00) |  | 1.46  (1.36, 1.57) | 17.29  (16.50, 18.11) |  | 0.05  (0.04, 0.07) | 5.58  (3.85, 7.83) |  | 0.05  (0.03, 0.06)* |  | -0.04  (-0.06, -0.03) | |
| Chile | 1.32  (1.25, 1.40) | 12.44  (11.80, 13.10) |  | 0.04  (0.03, 0.06) | 4.03  (2.80, 5.66) |  | 2.84  (2.69, 3.01) | 12.34  (11.69, 13.04) |  | 0.09  (0.06, 0.12) | 3.72  (2.55, 5.25) |  | -0.03  (-0.03, -0.03) |  | -0.26  (-0.27, -0.25) | |
| China | 199.26  (189.93, 208.54) | 20.52  (19.60, 21.42) |  | 5.58  (3.74, 7.97) | 5.89  (4.04, 8.39) |  | 407.29  (389.93, 425.41) | 21.11  (20.24, 22.02) |  | 10.48  (7.03, 15.08) | 5.72  (3.90, 8.15) |  | 0.10  (0.07, 0.13)* |  | -0.14  (-0.19, -0.09) | |
| Colombia | 4.45  (4.20, 4.72) | 20.69  (19.55, 21.96) |  | 0.11  (0.07, 0.16) | 5.26  (3.52, 7.58) |  | 10.75  (10.15, 11.38) | 20.54  (19.41, 21.73) |  | 0.26  (0.17, 0.38) | 5.00  (3.31, 7.27) |  | -0.02  (-0.02, -0.02) |  | -0.17  (-0.18, -0.16) | |
| Comoros | 0.05  (0.05, 0.05) | 17.28  (16.48, 18.09) |  | 0.00  (0.00, 0.00) | 5.36  (3.66, 7.60) |  | 0.10  (0.09, 0.10) | 16.84  (16.06, 17.65) |  | 0.00  (0.00, 0.00) | 4.99  (3.42, 7.04) |  | -0.08  (-0.08, -0.07) |  | -0.21  (-0.22, -0.20) | |
| Congo | 0.24  (0.23, 0.26) | 17.26  (16.53, 18.01) |  | 0.01  (0.01, 0.01) | 5.26  (3.61, 7.37) |  | 0.61  (0.58, 0.64) | 16.96  (16.21, 17.68) |  | 0.02  (0.01, 0.02) | 4.90  (3.38, 6.97) |  | -0.04  (-0.05, -0.04) |  | -0.22  (-0.23, -0.21) | |
| Cook Islands | 0.00  (0.00, 0.00) | 19.99  (19.27, 20.75) |  | 0.00  (0.00, 0.00) | 5.23  (3.51, 7.49) |  | 0.00  (0.00, 0.00) | 19.80  (19.11, 20.54) |  | 0.00  (0.00, 0.00) | 4.97  (3.33, 7.12) |  | -0.02  (-0.04, -0.01) |  | -0.15  (-0.17, -0.13) | |
| Costa Rica | 0.42  (0.40, 0.45) | 20.62  (19.50, 21.75) |  | 0.01  (0.01, 0.02) | 5.17  (3.47, 7.52) |  | 1.06  (1.00, 1.12) | 20.50  (19.35, 21.70) |  | 0.03  (0.02, 0.04) | 4.91  (3.27, 7.23) |  | -0.02  (-0.02, -0.01) |  | -0.17  (-0.18, -0.16) | |
| Cote d'Ivoire | 1.14  (1.06, 1.22) | 17.50  (16.74, 18.29) |  | 0.04  (0.02, 0.05) | 5.46  (3.75, 7.62) |  | 2.77  (2.60, 2.94) | 17.22  (16.51, 17.99) |  | 0.09  (0.06, 0.12) | 5.28  (3.61, 7.48) |  | -0.04  (-0.06, -0.03) |  | -0.10  (-0.12, -0.08) | |
| Croatia | 1.02  (0.98, 1.07) | 16.81  (16.07, 17.60) |  | 0.03  (0.02, 0.04) | 4.43  (2.99, 6.34) |  | 1.19  (1.13, 1.25) | 16.74  (16.00, 17.53) |  | 0.03  (0.02, 0.05) | 4.28  (2.89, 6.15) |  | -0.01  (-0.02, -0.01) |  | -0.13  (-0.14, -0.12) | |
| Cuba | 2.18  (2.06, 2.30) | 20.75  (19.62, 21.96) |  | 0.06  (0.04, 0.08) | 5.28  (3.53, 7.64) |  | 3.48  (3.28, 3.69) | 20.59  (19.44, 21.77) |  | 0.09  (0.06, 0.13) | 5.08  (3.36, 7.31) |  | -0.01  (-0.02, -0.01) |  | -0.11  (-0.12, -0.10) | |
| Cyprus | 0.09  (0.08, 0.09) | 10.44  (9.87, 11.02) |  | 0.00  (0.00, 0.00) | 2.72  (1.84, 3.97) |  | 0.19  (0.18, 0.20) | 10.23  (9.66, 10.79) |  | 0.00  (0.00, 0.01) | 2.44  (1.62, 3.60) |  | -0.07  (-0.07, -0.06) |  | -0.33  (-0.36, -0.29) | |
| Czech | 2.15  (2.05, 2.25) | 16.84  (16.10, 17.63) |  | 0.06  (0.04, 0.08) | 4.42  (2.98, 6.31) |  | 2.89  (2.76, 3.04) | 16.76  (16.03, 17.52) |  | 0.07  (0.05, 0.11) | 4.20  (2.84, 6.06) |  | 0.00  (-0.01, 0.01) |  | -0.09  (-0.12, -0.06) | |
| Denmark | 0.75  (0.70, 0.79) | 10.17  (9.61, 10.76) |  | 0.02  (0.01, 0.03) | 2.32  (1.52, 3.48) |  | 0.99  (0.92, 1.05) | 10.08  (9.53, 10.66) |  | 0.02  (0.01, 0.03) | 2.20  (1.41, 3.31) |  | -0.02  (-0.03, -0.01) |  | -0.12  (-0.16, -0.08) | |
| Djibouti | 0.04  (0.04, 0.05) | 17.55  (16.76, 18.34) |  | 0.00  (0.00, 0.00) | 5.43  (3.69, 7.69) |  | 0.15  (0.14, 0.15) | 17.14  (16.39, 17.91) |  | 0.00  (0.00, 0.01) | 5.03  (3.42, 7.11) |  | -0.08  (-0.09, -0.06) |  | -0.26  (-0.28, -0.24) | |
| Dominica | 0.01  (0.01, 0.01) | 20.66  (19.54, 21.88) |  | 0.00  (0.00, 0.00) | 5.26  (3.49, 7.58) |  | 0.02  (0.02, 0.02) | 20.58  (19.39, 21.90) |  | 0.00  (0.00, 0.00) | 4.97  (3.32, 7.27) |  | -0.01  (-0.02, -0.01) |  | -0.19  (-0.20, -0.18) | |
| Dominican Republic | 0.94  (0.89, 1.00) | 20.82  (19.67, 22.01) |  | 0.02  (0.02, 0.04) | 5.51  (3.66, 7.97) |  | 2.06  (1.95, 2.18) | 20.79  (19.69, 21.97) |  | 0.05  (0.03, 0.07) | 5.21  (3.46, 7.57) |  | 0.00  (-0.01, 0.00) |  | -0.21  (-0.22, -0.19) | |
| DR Congo | 3.68  (3.45, 3.92) | 17.14  (16.36, 17.91) |  | 0.12  (0.08, 0.17) | 5.48  (3.76, 7.63) |  | 8.37  (7.79, 8.91) | 16.61  (15.83, 17.38) |  | 0.27  (0.18, 0.38) | 5.18  (3.59, 7.30) |  | -0.10  (-0.13, -0.07) |  | -0.16  (-0.19, -0.14) | |
| Ecuador | 1.35  (1.27, 1.44) | 21.14  (19.93, 22.39) |  | 0.04  (0.02, 0.05) | 5.79  (3.90, 8.35) |  | 3.35  (3.16, 3.55) | 20.91  (19.74, 22.17) |  | 0.08  (0.06, 0.12) | 5.39  (3.66, 7.75) |  | -0.02  (-0.03, -0.01) |  | -0.15  (-0.21, -0.08) | |
| Egypt | 3.99  (3.74, 4.24) | 12.08  (11.41, 12.75) |  | 0.15  (0.11, 0.21) | 4.66  (3.29, 6.47) |  | 8.05  (7.57, 8.51) | 11.48  (10.82, 12.14) |  | 0.26  (0.18, 0.36) | 3.86  (2.70, 5.39) |  | -0.16  (-0.16, -0.15) |  | -0.58  (-0.61, -0.55) | |
| El Salvador | 0.70  (0.66, 0.74) | 20.57  (19.46, 21.74) |  | 0.02  (0.01, 0.03) | 5.34  (3.59, 7.70) |  | 1.22  (1.15, 1.29) | 20.45  (19.33, 21.67) |  | 0.03  (0.02, 0.04) | 5.02  (3.34, 7.31) |  | -0.03  (-0.03, -0.02) |  | -0.24  (-0.25, -0.22) | |
| Equatorial Guinea | 0.04  (0.04, 0.05) | 17.12  (16.30, 17.97) |  | 0.00  (0.00, 0.00) | 5.57  (3.85, 7.82) |  | 0.14  (0.13, 0.14) | 17.18  (16.51, 17.89) |  | 0.00  (0.00, 0.01) | 4.79  (3.29, 6.74) |  | 0.05  (0.02, 0.07)* |  | -0.56  (-0.59, -0.53) | |
| Eritrea | 0.26  (0.24, 0.28) | 17.07  (16.18, 17.94) |  | 0.01  (0.01, 0.01) | 5.42  (3.70, 7.65) |  | 0.67  (0.63, 0.72) | 16.90  (16.10, 17.67) |  | 0.02  (0.01, 0.03) | 5.04  (3.46, 7.11) |  | -0.04  (-0.06, -0.03) |  | -0.21  (-0.22, -0.20) | |
| Estonia | 0.32  (0.31, 0.34) | 16.75  (15.99, 17.54) |  | 0.01  (0.01, 0.01) | 4.60  (3.12, 6.53) |  | 0.36  (0.34, 0.38) | 16.71  (15.99, 17.51) |  | 0.01  (0.01, 0.01) | 4.38  (2.96, 6.29) |  | 0.00  (0.00, 0.01) |  | -0.13  (-0.14, -0.11) | |
| Eswatini | 0.08  (0.07, 0.08) | 17.79  (17.02, 18.61) |  | 0.00  (0.00, 0.00) | 5.71  (3.96, 8.04) |  | 0.14  (0.13, 0.14) | 17.58  (16.79, 18.38) |  | 0.00  (0.00, 0.01) | 5.26  (3.61, 7.40) |  | -0.04  (-0.05, -0.04) |  | -0.29  (-0.30, -0.27) | |
| Ethiopia | 4.77  (4.39, 5.17) | 17.35  (16.46, 18.27) |  | 0.17  (0.11, 0.24) | 5.83  (4.03, 8.21) |  | 10.54  (9.81, 11.36) | 17.42  (16.61, 18.23) |  | 0.35  (0.24, 0.50) | 5.53  (3.78, 7.80) |  | 0.04  (0.02, 0.05)* |  | -0.15  (-0.17, -0.13) | |
| Fiji | 0.10  (0.10, 0.11) | 19.89  (19.16, 20.65) |  | 0.00  (0.00, 0.00) | 5.39  (3.64, 7.67) |  | 0.16  (0.16, 0.17) | 19.75  (19.03, 20.51) |  | 0.00  (0.00, 0.01) | 5.13  (3.45, 7.33) |  | -0.02  (-0.03, 0.00) |  | -0.14  (-0.17, -0.11) | |
| Finland | 0.64  (0.61, 0.67) | 9.35  (8.90, 9.79) |  | 0.02  (0.01, 0.02) | 2.51  (1.70, 3.64) |  | 1.00  (0.92, 1.08) | 9.39  (8.76, 10.07) |  | 0.03  (0.02, 0.04) | 2.36  (1.57, 3.48) |  | 0.05  (0.03, 0.06)* |  | -0.08  (-0.14, -0.02) | |
| France | 7.95  (7.50, 8.44) | 10.42  (9.85, 11.01) |  | 0.22  (0.15, 0.31) | 2.76  (1.86, 3.96) |  | 11.68  (11.02, 12.41) | 10.31  (9.75, 10.87) |  | 0.32  (0.21, 0.46) | 2.59  (1.72, 3.79) |  | -0.02  (-0.03, -0.02) |  | -0.12  (-0.16, -0.08) | |
| Gabon | 0.12  (0.11, 0.12) | 17.59  (16.89, 18.33) |  | 0.00  (0.00, 0.01) | 5.29  (3.63, 7.48) |  | 0.23  (0.22, 0.24) | 17.09  (16.38, 17.81) |  | 0.01  (0.00, 0.01) | 4.79  (3.27, 6.8) |  | -0.09  (-0.09, -0.08) |  | -0.31  (-0.32, -0.29) | |
| Gambia | 0.09  (0.09, 0.10) | 17.28  (16.53, 18.08) |  | 0.00  (0.00, 0.00) | 5.57  (3.85, 7.81) |  | 0.23  (0.21, 0.24) | 16.93  (16.18, 17.71) |  | 0.01  (0.01, 0.01) | 5.27  (3.59, 7.44) |  | -0.07  (-0.08, -0.05) |  | -0.21  (-0.23, -0.18) | |
| Georgia | 1.01  (0.96, 1.06) | 16.75  (16.01, 17.55) |  | 0.03  (0.02, 0.04) | 4.59  (3.09, 6.56) |  | 0.88  (0.84, 0.92) | 16.67  (15.92, 17.46) |  | 0.02  (0.02, 0.03) | 4.46  (3.03, 6.36) |  | 0.00  (-0.02, 0.02) |  | -0.08  (-0.09, -0.06) | |
| Germany | 11.84  (11.14, 12.54) | 10.26  (9.71, 10.81) |  | 0.30  (0.20, 0.44) | 2.54  (1.67, 3.74) |  | 16.15  (15.15, 17.18) | 10.26  (9.70, 10.82) |  | 0.41  (0.27, 0.59) | 2.41  (1.58, 3.55) |  | 0.01  (0.00, 0.02)* |  | -0.06  (-0.12, 0.00) | |
| Ghana | 1.48  (1.39, 1.58) | 16.98  (16.21, 17.81) |  | 0.05  (0.03, 0.07) | 5.27  (3.64, 7.44) |  | 3.66  (3.47, 3.87) | 16.85  (16.15, 17.61) |  | 0.11  (0.07, 0.15) | 4.99  (3.40, 7.06) |  | -0.01  (-0.03, 0.01) |  | -0.16  (-0.19, -0.14) | |
| Greece | 1.48  (1.40, 1.57) | 10.53  (9.98, 11.12) |  | 0.04  (0.03, 0.06) | 2.96  (2.01, 4.25) |  | 2.01  (1.89, 2.15) | 10.35  (9.79, 10.92) |  | 0.06  (0.04, 0.08) | 2.72  (1.85, 3.95) |  | -0.05  (-0.05, -0.04) |  | -0.23  (-0.25, -0.21) | |
| Greenland | 0.01  (0.00, 0.01) | 12.95  (12.33, 13.63) |  | 0.00  (0.00, 0.00) | 4.05  (2.8, 5.65) |  | 0.01  (0.01, 0.01) | 12.89  (12.25, 13.59) |  | 0.00  (0.00, 0.00) | 3.92  (2.68, 5.53) |  | -0.01  (-0.02, -0.01) |  | -0.09  (-0.10, -0.08) | |
| Grenada | 0.01  (0.01, 0.02) | 20.79  (19.62, 21.95) |  | 0.00  (0.00, 0.00) | 5.43  (3.63, 7.85) |  | 0.02  (0.02, 0.03) | 20.71  (19.63, 21.94) |  | 0.00  (0.00, 0.00) | 5.08  (3.39, 7.40) |  | 0.00  (-0.01, 0.00) |  | -0.19  (-0.21, -0.16) | |
| Guam | 0.02  (0.02, 0.02) | 20.17  (19.45, 20.88) |  | 0.00  (0.00, 0.00) | 5.21  (3.50, 7.48) |  | 0.04  (0.04, 0.04) | 19.91  (19.21, 20.62) |  | 0.00  (0.00, 0.00) | 4.99  (3.32, 7.17) |  | -0.04  (-0.06, -0.02) |  | -0.16  (-0.19, -0.13) | |
| Guatemala | 0.94  (0.89, 1.00) | 20.80  (19.69, 21.97) |  | 0.02  (0.02, 0.04) | 5.50  (3.69, 7.91) |  | 2.63  (2.49, 2.78) | 20.51  (19.37, 21.68) |  | 0.06  (0.04, 0.09) | 5.10  (3.4,0 7.37) |  | -0.05  (-0.05, -0.04) |  | -0.26  (-0.27, -0.25) | |
| Guinea | 0.69  (0.65, 0.73) | 17.22  (16.44, 18.04) |  | 0.02  (0.02, 0.03) | 5.62  (3.84, 7.97) |  | 1.27  (1.19, 1.36) | 16.99  (16.22, 17.78) |  | 0.04  (0.03, 0.06) | 5.41  (3.70, 7.60) |  | -0.03  (-0.05, -0.01) |  | -0.10  (-0.12, -0.08) | |
| Guinea-Bissau | 0.10  (0.09, 0.10) | 17.07  (16.29, 17.90) |  | 0.00  (0.00, 0.00) | 5.54  (3.79, 7.79) |  | 0.18  (0.17, 0.20) | 16.82  (16.04, 17.61) |  | 0.01  (0.00, 0.01) | 5.32  (3.64, 7.48) |  | -0.04  (-0.06, -0.02) |  | -0.13  (-0.15, -0.11) | |
| Guyana | 0.10  (0.09, 0.10) | 20.62  (19.46, 21.89) |  | 0.00  (0.00, 0.00) | 5.33  (3.56, 7.73) |  | 0.14  (0.13, 0.15) | 20.59  (19.45, 21.80) |  | 0.00  (0.00, 0.00) | 5.08  (3.41, 7.31) |  | 0.00  (0.00, 0.00) |  | -0.14  (-0.15, -0.13) | |
| Haiti | 0.80  (0.76, 0.85) | 20.63  (19.50, 21.84) |  | 0.02  (0.01, 0.03) | 5.60  (3.77, 8.03) |  | 1.74  (1.64, 1.85) | 20.32  (19.17, 21.53) |  | 0.04  (0.03, 0.06) | 5.29  (3.56, 7.64) |  | -0.05  (-0.05, -0.04) |  | -0.18  (-0.19, -0.17) | |
| Honduras | 0.52  (0.50, 0.55) | 20.64  (19.49, 21.82) |  | 0.01  (0.01, 0.02) | 5.48  (3.68, 7.90) |  | 1.45  (1.36, 1.53) | 20.46  (19.34, 21.66) |  | 0.04  (0.02, 0.05) | 5.16  (3.46, 7.43) |  | -0.03  (-0.04, -0.03) |  | -0.21  (-0.22, -0.20) | |
| Hungary | 2.26  (2.15, 2.37) | 16.76  (15.99, 17.57) |  | 0.06  (0.04, 0.09) | 4.44  (2.98, 6.34) |  | 2.66  (2.53, 2.80) | 16.71  (15.92, 17.54) |  | 0.07  (0.05, 0.10) | 4.28  (2.89, 6.14) |  | -0.01  (-0.01, 0.00) |  | -0.10  (-0.12, -0.08) | |
| Iceland | 0.03  (0.03, 0.03) | 10.40  (9.82, 11.00) |  | 0.00  (0.00, 0.00) | 2.62  (1.74, 3.83) |  | 0.05  (0.05, 0.05) | 10.29  (9.71, 10.89) |  | 0.00  (0.00, 0.00) | 2.43  (1.61, 3.60) |  | -0.04  (-0.04, -0.03) |  | -0.21  (-0.24, -0.19) | |
| India | 108.23  (102.16, 114.57) | 19.14  (18.29, 20.04) |  | 3.26  (2.19, 4.63) | 5.74  (3.94, 8.09) |  | 241.61  (230.71, 252.83) | 19.29  (18.49, 20.14) |  | 6.88  (4.68, 9.81) | 5.61  (3.88, 7.95) |  | -0.03  (-0.05, 0.00) |  | -0.19  (-0.24, -0.14) | |
| Indonesia | 25.85  (24.59, 27.08) | 20.19  (19.42, 20.97) |  | 0.76  (0.51, 1.08) | 5.83  (3.99, 8.23) |  | 50.12  (48.11, 52.14) | 20.16  (19.44, 20.92) |  | 1.32  (0.88, 1.90) | 5.58  (3.79, 7.92) |  | 0.02  (0.00, 0.03)* |  | -0.07  (-0.10, -0.05) | |
| Iran | 3.87  (3.61, 4.13) | 12.54  (11.86, 13.26) |  | 0.16  (0.11, 0.22) | 4.88  (3.48, 6.80) |  | 9.18  (8.68, 9.69) | 11.67  (11.05, 12.31) |  | 0.29  (0.20, 0.41) | 3.92  (2.74, 5.49) |  | -0.26  (-0.27, -0.25) |  | -0.79  (-0.82, -0.76) | |
| Iraq | 1.13  (1.06, 1.20) | 12.32  (11.65, 13.00) |  | 0.04  (0.03, 0.06) | 4.63  (3.30, 6.44) |  | 3.08  (2.89, 3.26) | 11.44  (10.80, 12.09) |  | 0.10  (0.07, 0.14) | 3.77  (2.65, 5.31) |  | -0.24  (-0.25, -0.22) |  | -0.71  (-0.72, -0.70) | |
| Ireland | 0.41  (0.38, 0.43) | 10.36  (9.80, 10.94) |  | 0.01  (0.01, 0.02) | 2.64  (1.77, 3.86) |  | 0.70  (0.66, 0.74) | 10.32  (9.77, 10.89) |  | 0.02  (0.01, 0.02) | 2.43  (1.60, 3.61) |  | -0.01  (-0.02, -0.01) |  | -0.22  (-0.25, -0.20) | |
| Israel | 0.49  (0.47, 0.52) | 10.34  (9.77, 10.91) |  | 0.01  (0.01, 0.02) | 2.72  (1.84, 3.99) |  | 1.11  (1.04, 1.17) | 10.27  (9.70, 10.84) |  | 0.03  (0.02, 0.04) | 2.54  (1.68, 3.71) |  | -0.02  (-0.03, -0.02) |  | -0.16  (-0.19, -0.13) | |
| Italy | 8.68  (8.20, 9.20) | 10.71  (10.17, 11.30) |  | 0.26  (0.18, 0.36) | 3.17  (2.19, 4.47) |  | 12.35  (11.63, 13.12) | 10.59  (10.06, 11.17) |  | 0.38  (0.26, 0.53) | 2.99  (2.04, 4.24) |  | -0.04  (-0.04, -0.03) |  | -0.16  (-0.18, -0.15) | |
| Jamaica | 0.38  (0.36, 0.40) | 20.75  (19.60, 21.91) |  | 0.01  (0.01, 0.01) | 5.36  (3.53, 7.77) |  | 0.62  (0.58, 0.65) | 20.54  (19.43, 21.77) |  | 0.02  (0.01, 0.02) | 5.04  (3.36, 7.38) |  | -0.03  (-0.04, -0.03) |  | -0.20  (-0.21, -0.20) | |
| Japan | 21.11  (20.06, 22.21) | 12.97  (12.35, 13.62) |  | 0.61  (0.42, 0.88) | 3.86  (2.62, 5.49) |  | 35.33  (33.35, 37.38) | 12.87  (12.24, 13.53) |  | 1.11  (0.76, 1.56) | 3.67  (2.50, 5.26) |  | -0.03  (-0.03, -0.02) |  | -0.12  (-0.14, -0.10) | |
| Jordan | 0.21  (0.19, 0.22) | 12.05  (11.41, 12.74) |  | 0.01  (0.01, 0.01) | 4.48  (3.18, 6.24) |  | 0.88  (0.82, 0.93) | 11.33  (10.68, 11.98) |  | 0.03  (0.02, 0.04) | 3.69  (2.56, 5.18) |  | -0.21  (-0.22, -0.20) |  | -0.67  (-0.69, -0.66) | |
| Kazakhstan | 2.31  (2.20, 2.42) | 16.86  (16.10, 17.65) |  | 0.06  (0.04, 0.09) | 4.67  (3.15, 6.66) |  | 3.05  (2.91, 3.20) | 16.82  (16.07, 17.62) |  | 0.08  (0.05, 0.11) | 4.46  (3.01, 6.38) |  | 0.00  (-0.01, 0.02) |  | -0.14  (-0.16, -0.13) | |
| Kenya | 2.55  (2.37, 2.74) | 21.50  (20.38, 22.71) |  | 0.09  (0.06, 0.13) | 6.98  (4.84, 9.79) |  | 6.70  (6.27, 7.15) | 21.35  (20.26, 22.50) |  | 0.22  (0.15, 0.31) | 6.77  (4.65, 9.55) |  | -0.02  (-0.04, -0.01) |  | -0.09  (-0.10, -0.07) | |
| Kiribati | 0.01  (0.01, 0.01) | 19.69  (18.94, 20.50) |  | 0.00  (0.00, 0.00) | 5.50  (3.75, 7.78) |  | 0.02  (0.02, 0.02) | 19.58  (18.80, 20.37) |  | 0.00  (0.00, 0.00) | 5.45  (3.71, 7.69) |  | -0.01  (-0.02, 0.01) |  | 0.01  (-0.01, 0.03) | |
| Kuwait | 0.12  (0.11, 0.12) | 12.29  (11.65, 12.96) |  | 0.00  (0.00, 0.01) | 4.28  (2.99, 6.01) |  | 0.41  (0.39, 0.44) | 11.58  (10.94, 12.21) |  | 0.01  (0.01, 0.02) | 3.58  (2.47, 5.07) |  | -0.24  (-0.25, -0.22) |  | -0.67  (-0.70, -0.65) | |
| Kyrgyzstan | 0.55  (0.53, 0.58) | 16.70  (15.95, 17.50) |  | 0.02  (0.01, 0.02) | 4.78  (3.24, 6.81) |  | 0.86  (0.82, 0.91) | 16.54  (15.79, 17.38) |  | 0.02  (0.02, 0.03) | 4.67  (3.16, 6.66) |  | -0.03  (-0.04, -0.01) |  | -0.05  (-0.06, -0.04) | |
| Laos | 0.53  (0.50, 0.56) | 20.22  (19.41, 21.08) |  | 0.02  (0.01, 0.02) | 6.12  (4.15, 8.70) |  | 1.12  (1.07, 1.17) | 20.21  (19.47, 20.97) |  | 0.03  (0.02, 0.05) | 5.76  (3.91, 8.19) |  | 0.01  (0.00, 0.03)* |  | -0.19  (-0.20, -0.17) | |
| Latvia | 0.56  (0.53, 0.58) | 16.73  (15.98, 17.52) |  | 0.02  (0.01, 0.02) | 4.57  (3.09, 6.55) |  | 0.54  (0.51, 0.56) | 16.67  (15.96, 17.41) |  | 0.01  (0.01, 0.02) | 4.38  (2.97, 6.27) |  | 0.00  (-0.01, 0.01) |  | -0.11  (-0.13, -0.10) | |
| Lebanon | 0.29  (0.27, 0.31) | 12.16  (11.48, 12.84) |  | 0.01  (0.01, 0.01) | 4.54  (3.21, 6.32) |  | 0.60  (0.56, 0.63) | 11.34  (10.71, 12.00) |  | 0.02  (0.01, 0.03) | 3.66  (2.56, 5.15) |  | -0.25  (-0.25, -0.24) |  | -0.77  (-0.79, -0.76) | |
| Lesotho | 0.20  (0.19, 0.22) | 17.45  (16.60, 18.31) |  | 0.01  (0.00, 0.01) | 5.79  (3.98, 8.09) |  | 0.27  (0.26, 0.29) | 17.42  (16.64, 18.25) |  | 0.01  (0.01, 0.01) | 5.38  (3.76, 7.55) |  | -0.01  (-0.01, 0.00) |  | -0.26  (-0.27, -0.25) | |
| Liberia | 0.22  (0.21, 0.24) | 17.10  (16.33, 17.94) |  | 0.01  (0.00, 0.01) | 5.41  (3.67, 7.59) |  | 0.50  (0.47, 0.54) | 16.74  (15.94, 17.56) |  | 0.02  (0.01, 0.02) | 5.23  (3.63, 7.37) |  | -0.01  (-0.03, 0.00) |  | -0.11  (-0.14, -0.08) | |
| Libya | 0.28  (0.26, 0.29) | 12.39  (11.73, 13.05) |  | 0.01  (0.01, 0.01) | 4.58  (3.25, 6.37) |  | 0.65  (0.61, 0.69) | 11.30  (10.68, 11.95) |  | 0.02  (0.01, 0.03) | 3.64  (2.54, 5.12) |  | -0.30  (-0.32, -0.29) |  | -0.78  (-0.82, -0.73) | |
| Lithuania | 0.72  (0.69, 0.76) | 16.76  (15.99, 17.54) |  | 0.02  (0.01, 0.03) | 4.59  (3.10, 6.55) |  | 0.78  (0.74, 0.82) | 16.68  (15.94, 17.47) |  | 0.02  (0.01, 0.03) | 4.35  (2.94, 6.19) |  | -0.01  (-0.02, 0.00) |  | -0.18  (-0.19, -0.16) | |
| Luxembourg | 0.05  (0.05, 0.06) | 10.24  (9.65, 10.81) |  | 0.00  (0.00, 0.00) | 2.39  (1.57, 3.56) |  | 0.09  (0.09, 0.10) | 10.26  (9.70, 10.86) |  | 0.00  (0.00, 0.00) | 2.28  (1.46, 3.42) |  | 0.01  (0.01, 0.02)* |  | -0.09  (-0.12, -0.07) | |
| Madagascar | 1.30  (1.21, 1.41) | 18.44  (17.51, 19.49) |  | 0.05  (0.03, 0.07) | 6.14  (4.15, 8.76) |  | 2.92  (2.71, 3.15) | 17.93  (17.04, 18.89) |  | 0.10  (0.06, 0.14) | 5.83  (3.94, 8.26) |  | -0.11  (-0.14, -0.07) |  | -0.19  (-0.27, -0.12) | |
| Malawi | 0.89  (0.83, 0.96) | 17.08  (16.22, 17.96) |  | 0.03  (0.02, 0.04) | 5.42  (3.75, 7.67) |  | 1.74  (1.61, 1.86) | 16.81  (16.01, 17.63) |  | 0.05  (0.04, 0.08) | 5.10  (3.47, 7.25) |  | -0.04  (-0.05, -0.03) |  | -0.19  (-0.20, -0.17) | |
| Malaysia | 2.49  (2.37, 2.61) | 20.40  (19.64, 21.19) |  | 0.07  (0.05, 0.10) | 5.73  (3.94, 8.22) |  | 6.01  (5.79, 6.25) | 20.15  (19.43, 20.91) |  | 0.15  (0.10, 0.22) | 5.31  (3.59, 7.61) |  | -0.03  (-0.05, -0.01) |  | -0.23  (-0.26, -0.21) | |
| Maldives | 0.03  (0.02, 0.03) | 20.64  (19.86, 21.45) |  | 0.00  (0.00, 0.00) | 6.16  (4.20, 8.81) |  | 0.09  (0.09, 0.09) | 20.41  (19.68, 21.18) |  | 0.00  (0.00, 0.00) | 5.65  (3.81, 8.05) |  | -0.02  (-0.04, 0.00) |  | -0.26  (-0.28, -0.23) | |
| Mali | 0.89  (0.84, 0.95) | 17.04  (16.25, 17.87) |  | 0.03  (0.02, 0.04) | 5.61  (3.85, 7.86) |  | 2.09  (1.95, 2.23) | 17.15  (16.38, 17.97) |  | 0.07  (0.05, 0.10) | 5.54  (3.79, 7.79) |  | 0.05  (0.03, 0.06)* |  | -0.01  (-0.03, 0.01) | |
| Malta | 0.04  (0.04, 0.05) | 10.34  (9.77, 10.93) |  | 0.00  (0.00, 0.00) | 2.71  (1.84, 3.96) |  | 0.08  (0.08, 0.09) | 10.31  (9.74, 10.89) |  | 0.00  (0.00, 0.00) | 2.49  (1.67, 3.68) |  | -0.01  (-0.02, -0.01) |  | -0.23  (-0.26, -0.21) | |
| Marshall Islands | 0.00  (0.00, 0.01) | 19.91  (19.14, 20.69) |  | 0.00  (0.00, 0.00) | 5.60  (3.80, 7.98) |  | 0.01  (0.01, 0.01) | 19.69  (18.94, 20.44) |  | 0.00  (0.00, 0.00) | 5.31  (3.61, 7.50) |  | -0.03  (-0.04, -0.01) |  | -0.16  (-0.18, -0.14) | |
| Mauritania | 0.22  (0.21, 0.23) | 17.18  (16.44, 17.98) |  | 0.01  (0.00, 0.01) | 5.39  (3.71, 7.63) |  | 0.45  (0.43, 0.48) | 17.03  (16.30, 17.79) |  | 0.01  (0.01, 0.02) | 5.17  (3.52, 7.32) |  | -0.01  (-0.03, 0.00) |  | -0.11  (-0.12, -0.09) | |
| Mauritius | 0.18  (0.17, 0.19) | 20.32  (19.55, 21.11) |  | 0.01  (0.00, 0.01) | 5.74  (3.92, 8.18) |  | 0.33  (0.32, 0.34) | 20.15  (19.45, 20.89) |  | 0.01  (0.01, 0.01) | 5.35  (3.60, 7.70) |  | -0.02  (-0.04, -0.01) |  | -0.23  (-0.24, -0.21) | |
| Mexico | 11.05  (10.46, 11.68) | 20.95  (19.86, 22.10) |  | 0.29  (0.19, 0.41) | 5.44  (3.66, 7.86) |  | 25.56  (24.20, 27.07) | 20.78  (19.67, 21.95) |  | 0.62  (0.42, 0.91) | 5.20  (3.49, 7.52) |  | -0.03  (-0.04, -0.03) |  | -0.17  (-0.17, -0.16) | |
| Micronesia | 0.01  (0.01, 0.01) | 19.81  (19.05, 20.60) |  | 0.00  (0.00, 0.00) | 5.51  (3.75, 7.84) |  | 0.02  (0.02, 0.02) | 19.62  (18.90, 20.37) |  | 0.00  (0.00, 0.00) | 5.28  (3.57, 7.51) |  | -0.02  (-0.04, -0.01) |  | -0.14  (-0.15, -0.12) | |
| Moldova | 0.74  (0.71, 0.78) | 16.69  (15.94, 17.51) |  | 0.02  (0.01, 0.03) | 4.72  (3.22, 6.71) |  | 0.87  (0.83, 0.92) | 16.48  (15.74, 17.31) |  | 0.02  (0.02, 0.03) | 4.53  (3.07, 6.43) |  | -0.04  (-0.05, -0.02) |  | -0.14  (-0.15, -0.12) | |
| Monaco | 0.01  (0.01, 0.01) | 10.53  (9.97, 11.09) |  | 0.00  (0.00, 0.00) | 2.57  (1.69, 3.79) |  | 0.01  (0.01, 0.01) | 10.45  (9.87, 11.04) |  | 0.00  (0.00, 0.00) | 2.43  (1.60, 3.62) |  | -0.03  (-0.03, -0.03) |  | -0.15  (-0.17, -0.13) | |
| Mongolia | 0.21  (0.20, 0.22) | 16.79  (16.04, 17.60) |  | 0.01  (0.00, 0.01) | 4.84  (3.28, 6.90) |  | 0.47  (0.45, 0.49) | 16.81  (16.08, 17.63) |  | 0.01  (0.01, 0.02) | 4.62  (3.10, 6.53) |  | 0.01  (0.00, 0.02)* |  | -0.15  (-0.16, -0.13) | |
| Montenegro | 0.11  (0.10, 0.11) | 16.76  (15.99, 17.57) |  | 0.00  (0.00, 0.00) | 4.42  (2.96, 6.37) |  | 0.15  (0.14, 0.15) | 16.67  (15.92, 17.47) |  | 0.00  (0.00, 0.01) | 4.29  (2.90, 6.18) |  | -0.02  (-0.02, -0.01) |  | -0.12  (-0.13, -0.11) | |
| Morocco | 1.87  (1.75, 2.00) | 12.19  (11.48, 12.93) |  | 0.08  (0.05, 0.10) | 4.83  (3.45, 6.63) |  | 3.75  (3.52, 3.97) | 11.55  (10.90, 12.23) |  | 0.12  (0.09, 0.17) | 4.00  (2.79, 5.58) |  | -0.19  (-0.20, -0.19) |  | -0.65  (-0.66, -0.64) | |
| Mozambique | 1.30  (1.21, 1.40) | 16.87  (15.94, 17.74) |  | 0.04  (0.03, 0.06) | 5.49  (3.80, 7.71) |  | 2.69  (2.51, 2.89) | 16.99  (16.21, 17.81) |  | 0.09  (0.06, 0.12) | 5.19  (3.54, 7.35) |  | 0.05  (0.04, 0.06)* |  | -0.17  (-0.19, -0.16) | |
| Myanmar | 5.80  (5.48, 6.13) | 20.31  (19.44, 21.22) |  | 0.18  (0.12, 0.26) | 6.25  (4.17, 8.96) |  | 10.48  (10.06, 10.93) | 20.45  (19.72, 21.27) |  | 0.30  (0.20, 0.43) | 5.98  (4.05, 8.53) |  | 0.05  (0.03, 0.06)* |  | -0.15  (-0.17, -0.12) | |
| Namibia | 0.16  (0.15, 0.17) | 17.81  (17.00, 18.66) |  | 0.01  (0.00, 0.01) | 5.63  (3.93, 7.91) |  | 0.31  (0.29, 0.33) | 17.61  (16.86, 18.39) |  | 0.01  (0.01, 0.01) | 5.24  (3.61, 7.42) |  | -0.03  (-0.04, -0.03) |  | -0.24  (-0.24, -0.23) | |
| Nauru | 0.00  (0.00, 0.00) | 20.18  (19.47, 20.92) |  | 0.00  (0.00, 0.00) | 5.44  (3.65, 7.74) |  | 0.00  (0.00, 0.00) | 19.81  (19.07, 20.53) |  | 0.00  (0.00, 0.00) | 5.21  (3.52, 7.42) |  | -0.06  (-0.08, -0.04) |  | -0.12  (-0.15, -0.09) | |
| Nepal | 2.11  (1.98, 2.24) | 18.19  (17.35, 19.10) |  | 0.07  (0.04, 0.09) | 5.45  (3.74, 7.70) |  | 4.40  (4.18, 4.63) | 17.84  (17.00, 18.71) |  | 0.12  (0.08, 0.17) | 4.83  (3.27, 6.86) |  | -0.08  (-0.09, -0.07) |  | -0.45  (-0.47, -0.44) | |
| Netherlands | 1.94  (1.83, 2.06) | 10.29  (9.72, 10.88) |  | 0.05  (0.03, 0.07) | 2.50  (1.65, 3.73) |  | 2.97  (2.80, 3.16) | 10.23  (9.67, 10.81) |  | 0.07  (0.05, 0.11) | 2.34  (1.53, 3.51) |  | -0.01  (-0.02, 0.00) |  | -0.10  (-0.16, -0.05) | |
| New Zealand | 0.50  (0.48, 0.53) | 13.19  (12.48, 13.94) |  | 0.01  (0.01, 0.02) | 3.85  (2.65, 5.46) |  | 0.90  (0.84, 0.96) | 13.03  (12.31, 13.82) |  | 0.03  (0.02, 0.04) | 3.66  (2.48, 5.20) |  | -0.04  (-0.04, -0.04) |  | -0.13  (-0.15, -0.11) | |
| Nicaragua | 0.41  (0.38, 0.43) | 20.64  (19.48, 21.86) |  | 0.01  (0.01, 0.02) | 5.44  (3.64, 7.88) |  | 1.05  (0.99, 1.11) | 20.44  (19.33, 21.63) |  | 0.03  (0.02, 0.04) | 5.11  (3.44, 7.45) |  | -0.03  (-0.03, -0.02) |  | -0.21  (-0.22, -0.20) | |
| Niger | 0.71  (0.66, 0.76) | 17.05  (16.21, 17.92) |  | 0.03  (0.02, 0.04) | 5.68  (3.87, 7.98) |  | 1.99  (1.84, 2.15) | 17.04  (16.21, 17.89) |  | 0.07  (0.05, 0.10) | 5.69  (3.85, 7.93) |  | 0.03  (0.01, 0.05)* |  | 0.05  (0.03, 0.08)* | |
| Nigeria | 10.09  (9.46, 10.79) | 17.25  (16.46, 18.08) |  | 0.39  (0.26, 0.55) | 6.21  (4.26, 8.62) |  | 22.66  (21.16, 24.25) | 17.07  (16.33, 17.84) |  | 0.86  (0.58, 1.21) | 5.99  (4.11, 8.31) |  | -0.03  (-0.05, 0.00) |  | -0.19  (-0.25, -0.13) | |
| Niue | 0.00  (0.00, 0.00) | 19.88  (19.16, 20.64) |  | 0.00  (0.00, 0.00) | 5.29  (3.56, 7.54) |  | 0.00  (0.00, 0.00) | 19.73  (19.01, 20.46) |  | 0.00  (0.00, 0.00) | 5.02  (3.39, 7.17) |  | -0.02  (-0.03, 0.00) |  | -0.18  (-0.20, -0.16) | |
| North Korea | 3.56  (3.41, 3.71) | 19.81  (19.06, 20.62) |  | 0.09  (0.06, 0.14) | 5.45  (3.70, 7.82) |  | 6.14  (5.90, 6.39) | 19.48  (18.72, 20.26) |  | 0.16  (0.11, 0.23) | 5.26  (3.53, 7.55) |  | -0.03  (-0.05, 0.00) |  | -0.09  (-0.11, -0.06) | |
| North Macedonia | 0.33  (0.31, 0.34) | 16.80  (16.07, 17.59) |  | 0.01  (0.01, 0.01) | 4.55  (3.07, 6.49) |  | 0.50  (0.47, 0.52) | 16.71  (15.97, 17.52) |  | 0.01  (0.01, 0.02) | 4.37  (2.94, 6.28) |  | -0.02  (-0.03, -0.01) |  | -0.15  (-0.16, -0.14) | |
| Northern Mariana Islands | 0.01  (0.01, 0.01) | 20.21  (19.46, 20.98) |  | 0.00  (0.00, 0.00) | 5.19  (3.49, 7.45) |  | 0.01  (0.01, 0.01) | 19.88  (19.19, 20.62) |  | 0.00  (0.00, 0.00) | 5.05  (3.36, 7.21) |  | -0.04  (-0.05, -0.02) |  | -0.04  (-0.07, -0.02) | |
| Norway | 0.74  (0.69, 0.80) | 11.90  (11.18, 12.71) |  | 0.02  (0.01, 0.03) | 3.14  (2.10, 4.55) |  | 1.00  (0.93, 1.07) | 11.73  (11.03, 12.50) |  | 0.03  (0.02, 0.04) | 2.98  (1.98, 4.34) |  | -0.05  (-0.07, -0.04) |  | -0.18  (-0.20, -0.17) | |
| Oman | 0.12  (0.11, 0.13) | 12.67  (12.10, 13.29) |  | 0.00  (0.00, 0.01) | 4.79  (3.42, 6.55) |  | 0.34  (0.31, 0.36) | 11.65  (11.04, 12.30) |  | 0.01  (0.01, 0.01) | 3.66  (2.51, 5.17) |  | -0.20  (-0.25, -0.15) |  | -0.75  (-0.85, -0.64) | |
| Pakistan | 12.35  (11.70, 13.03) | 18.41  (17.61, 19.28) |  | 0.39  (0.26, 0.54) | 5.48  (3.75, 7.71) |  | 25.83  (24.48, 27.19) | 18.38  (17.60, 19.20) |  | 0.74  (0.50, 1.05) | 5.21  (3.58, 7.34) |  | -0.03  (-0.03, -0.02) |  | -0.21  (-0.23, -0.20) | |
| Palau | 0.00  (0.00, 0.00) | 19.97  (19.24, 20.71) |  | 0.00  (0.00, 0.00) | 5.27  (3.55, 7.58) |  | 0.00  (0.00, 0.00) | 19.84  (19.13, 20.53) |  | 0.00  (0.00, 0.00) | 5.04  (3.40, 7.24) |  | -0.01  (-0.03, 0.00) |  | -0.12  (-0.14, -0.11) | |
| Palestine | 0.12  (0.11, 0.13) | 12.03  (11.29, 12.73) |  | 0.01  (0.00, 0.01) | 4.80  (3.43, 6.62) |  | 0.32  (0.30, 0.34) | 11.28  (10.67, 11.94) |  | 0.01  (0.01, 0.01) | 3.82  (2.65, 5.33) |  | -0.24  (-0.24, -0.23) |  | -0.80  (-0.81, -0.79) | |
| Panama | 0.35  (0.33, 0.37) | 20.64  (19.51, 21.84) |  | 0.01  (0.01, 0.01) | 5.16  (3.43, 7.50) |  | 0.86  (0.81, 0.91) | 20.62  (19.48, 21.78) |  | 0.02  (0.01, 0.03) | 4.93  (3.27, 7.16) |  | 0.00  (0.00, 0.00) |  | -0.15  (-0.16, -0.15) | |
| Papua New Guinea | 0.51  (0.48, 0.53) | 20.01  (19.24, 20.79) |  | 0.01  (0.01, 0.02) | 5.76  (3.90, 8.18) |  | 1.32  (1.25, 1.39) | 19.91  (19.14, 20.70) |  | 0.04  (0.02, 0.05) | 5.60  (3.84, 7.93) |  | -0.01  (-0.03, 0.01) |  | -0.06  (-0.08, -0.05) | |
| Paraguay | 0.55  (0.52, 0.58) | 21.08  (20.01, 22.14) |  | 0.01  (0.01, 0.02) | 5.50  (3.64, 7.97) |  | 1.27  (1.20, 1.34) | 20.92  (19.85, 22.05) |  | 0.03  (0.02, 0.04) | 5.19  (3.42, 7.56) |  | -0.03  (-0.03, -0.02) |  | -0.19  (-0.20, -0.19) | |
| Peru | 2.89  (2.73, 3.08) | 20.55  (19.28, 21.86) |  | 0.07  (0.05, 0.10) | 5.03  (3.38, 7.28) |  | 6.80  (6.41, 7.22) | 20.54  (19.33, 21.86) |  | 0.16  (0.11, 0.24) | 4.88  (3.26, 7.17) |  | 0.01  (0.00, 0.01)* |  | -0.09  (-0.10, -0.09) | |
| Philippines | 8.28  (7.85, 8.71) | 20.52  (19.71, 21.32) |  | 0.26  (0.17, 0.37) | 6.12  (4.20, 8.69) |  | 18.80  (18.00, 19.59) | 20.36  (19.60, 21.12) |  | 0.53  (0.36, 0.76) | 5.85  (3.98, 8.28) |  | -0.01  (-0.02, 0.01) |  | -0.09  (-0.12, -0.06) | |
| Poland | 7.26  (6.93, 7.59) | 17.08  (16.35, 17.88) |  | 0.20  (0.14, 0.29) | 4.88  (3.33, 6.93) |  | 10.17  (9.70, 10.66) | 17.08  (16.34, 17.87) |  | 0.28  (0.19, 0.40) | 4.69  (3.19, 6.68) |  | 0.00  (0.00, 0.00) |  | -0.11  (-0.13, -0.10) | |
| Portugal | 1.36  (1.29, 1.44) | 10.45  (9.88, 11.03) |  | 0.04  (0.02, 0.05) | 2.78  (1.90, 4.01) |  | 2.05  (1.93, 2.18) | 10.30  (9.75, 10.86) |  | 0.05  (0.04, 0.08) | 2.55  (1.70, 3.75) |  | -0.05  (-0.06, -0.05) |  | -0.24  (-0.27, -0.22) | |
| Puerto Rico | 0.75  (0.71, 0.79) | 20.82  (19.70, 22.04) |  | 0.02  (0.01, 0.03) | 5.13  (3.41, 7.52) |  | 1.17  (1.10, 1.25) | 20.69  (19.54, 21.89) |  | 0.03  (0.02, 0.04) | 4.87  (3.22, 7.16) |  | -0.02  (-0.02, -0.01) |  | -0.17  (-0.17, -0.16) | |
| Qatar | 0.03  (0.03, 0.03) | 12.60(11.95, 13.26) |  | 0.00  (0.00, 0.00) | 4.44  (3.09, 6.14) |  | 0.23  (0.22, 0.25) | 11.93  (11.28, 12.55) |  | 0.01  (0.00, 0.01) | 3.63  (2.52, 5.12) |  | -0.17  (-0.18, -0.16) |  | -0.66  (-0.69, -0.63) | |
| Romania | 4.51  (4.30, 4.73) | 16.81  (16.07, 17.60) |  | 0.12  (0.08, 0.17) | 4.55  (3.06, 6.46) |  | 5.19  (4.95, 5.44) | 16.77  (16.03, 17.53) |  | 0.14  (0.09, 0.20) | 4.37  (2.93, 6.29) |  | 0.00  (-0.01, 0.01) |  | -0.11  (-0.13, -0.10) | |
| Russia | 29.63  (28.28, 31.00) | 17.02  (16.28, 17.82) |  | 0.81  (0.54, 1.14) | 4.79  (3.25, 6.78) |  | 35.69  (34.10, 37.39) | 16.89  (16.16, 17.67) |  | 0.96  (0.65, 1.37) | 4.60  (3.13, 6.53) |  | -0.01  (-0.02, -0.01) |  | -0.11  (-0.12, -0.10) | |
| Rwanda | 0.67  (0.62, 0.71) | 17.05  (16.21, 17.88) |  | 0.02  (0.01, 0.03) | 5.33  (3.66, 7.52) |  | 1.38  (1.30, 1.47) | 16.93  (16.15, 17.72) |  | 0.04  (0.03, 0.06) | 5.02  (3.44, 7.16) |  | 0.00  (-0.02, 0.01) |  | -0.18  (-0.19, -0.16) | |
| Saint Kitts and Nevis | 0.01  (0.01, 0.01) | 20.77  (19.61, 22.01) |  | 0.00  (0.00, 0.00) | 5.22  (3.46, 7.61) |  | 0.01  (0.01, 0.02) | 20.74  (19.61, 21.93) |  | 0.00  (0.00, 0.00) | 4.97  (3.32, 7.33) |  | -0.01  (-0.01, 0.00) |  | -0.16  (-0.16, -0.15) | |
| Saint Lucia | 0.02  (0.02, 0.02) | 20.79  (19.67, 22.00) |  | 0.00  (0.00, 0.00) | 5.34  (3.58, 7.78) |  | 0.04  (0.04, 0.05) | 20.67  (19.55, 21.83) |  | 0.00  (0.00, 0.00) | 5.04  (3.34, 7.33) |  | -0.02  (-0.02, -0.02) |  | -0.18  (-0.19, -0.16) | |
| Saint Vincent and the Grenadines | 0.02  (0.02, 0.02) | 20.78  (19.61, 22.05) |  | 0.00  (0.00, 0.00) | 5.42  (3.63, 7.80) |  | 0.03  (0.03, 0.03) | 20.70  (19.54, 21.87) |  | 0.00  (0.00, 0.00) | 5.12  (3.41, 7.50) |  | -0.01  (-0.01, 0.00) |  | -0.17  (-0.18, -0.16) | |
| Samoa | 0.02  (0.02, 0.02) | 19.81  (19.04, 20.58) |  | 0.00  (0.00, 0.00) | 5.40  (3.65, 7.66) |  | 0.03  (0.03, 0.03) | 19.79  (19.06, 20.52) |  | 0.00  (0.00, 0.00) | 5.32  (3.58, 7.59) |  | 0.02  (0.00, 0.03)* |  | -0.02  (-0.04, 0.00) | |
| San Marino | 0.00  (0.00, 0.00) | 10.43  (9.85, 11.01) |  | 0.00  (0.00, 0.00) | 2.58  (1.72, 3.82) |  | 0.01  (0.01, 0.01) | 10.28  (9.72, 10.86) |  | 0.00  (0.00, 0.00) | 2.42  (1.60, 3.59) |  | -0.04  (-0.05, -0.03) |  | -0.16  (-0.19, -0.13) | |
| Sao Tome and Principe | 0.01  (0.01, 0.01) | 17.24  (16.49, 18.04) |  | 0.00  (0.00, 0.00) | 5.44  (3.70, 7.71) |  | 0.02  (0.02, 0.03) | 16.99  (16.29, 17.75) |  | 0.00  (0.00, 0.00) | 5.12  (3.52, 7.25) |  | -0.04  (-0.06, -0.02) |  | -0.22  (-0.24, -0.20) | |
| Saudi Arabia | 1.04  (0.97, 1.10) | 12.85  (12.16, 13.53) |  | 0.04  (0.03, 0.06) | 4.89  (3.47, 6.74) |  | 2.96  (2.78, 3.15) | 11.66  (11.00, 12.29) |  | 0.08  (0.06, 0.12) | 3.63  (2.52, 5.10) |  | -0.33  (-0.36, -0.30) |  | -0.99  (-1.05, -0.93) | |
| Senegal | 0.75  (0.70, 0.79) | 17.20  (16.44, 18.03) |  | 0.03  (0.02, 0.04) | 5.49  (3.80, 7.71) |  | 1.68  (1.58, 1.78) | 17.05  (16.32, 17.86) |  | 0.05  (0.04, 0.08) | 5.32  (3.67, 7.45) |  | -0.01  (-0.03, 0.00) |  | -0.09  (-0.10, -0.07) | |
| Serbia | 1.86  (1.77, 1.94) | 16.79  (16.03, 17.58) |  | 0.05  (0.03, 0.07) | 4.55  (3.07, 6.57) |  | 2.24  (2.13, 2.36) | 16.66  (15.93, 17.45) |  | 0.06  (0.04, 0.08) | 4.32  (2.92, 6.22) |  | -0.02  (-0.03, -0.01) |  | -0.17  (-0.18, -0.17) | |
| Seychelles | 0.01  (0.01, 0.01) | 20.42  (19.68, 21.20) |  | 0.00  (0.00, 0.00) | 5.73  (3.90, 8.18) |  | 0.02  (0.02, 0.02) | 20.24  (19.52, 21.00) |  | 0.00  (0.00, 0.00) | 5.36  (3.60, 7.64) |  | -0.02  (-0.04, 0.00) |  | -0.19  (-0.22, -0.16) | |
| Sierra Leone | 0.43  (0.40, 0.45) | 18.61  (17.56, 19.62) |  | 0.01  (0.01, 0.02) | 5.46  (3.77, 7.74) |  | 0.90  (0.84, 0.96) | 18.34  (17.35, 19.39) |  | 0.03  (0.02, 0.04) | 5.22  (3.55, 7.43) |  | -0.04  (-0.05, -0.02) |  | -0.13  (-0.15, -0.10) | |
| Singapore | 0.33  (0.31, 0.34) | 12.94  (12.30, 13.61) |  | 0.01  (0.01, 0.01) | 3.86  (2.64, 5.53) |  | 0.99  (0.94, 1.05) | 12.85  (12.20, 13.51) |  | 0.03  (0.02, 0.04) | 3.56  (2.41, 5.20) |  | -0.01  (-0.02, 0.00) |  | -0.23  (-0.25, -0.21) | |
| Slovakia | 0.97  (0.93, 1.02) | 16.80  (16.06, 17.57) |  | 0.03  (0.02, 0.04) | 4.48  (3.03, 6.39) |  | 1.36  (1.30, 1.43) | 16.75  (16.01, 17.51) |  | 0.03  (0.02, 0.05) | 4.26  (2.87, 6.15) |  | 0.00  (-0.01, 0.01) |  | -0.15  (-0.17, -0.13) | |
| Slovenia | 0.39  (0.37, 0.41) | 16.64  (15.9, 17.43) |  | 0.01  (0.01, 0.01) | 4.09  (2.74, 5.90) |  | 0.58  (0.55, 0.61) | 16.58  (15.79, 17.35) |  | 0.01  (0.01, 0.02) | 3.93  (2.63, 5.73) |  | -0.01  (-0.01, -0.01) |  | -0.09  (-0.11, -0.06) | |
| Solomon Islands | 0.04  (0.04, 0.04) | 19.94  (19.15, 20.76) |  | 0.00  (0.00, 0.00) | 5.81  (3.95, 8.26) |  | 0.08  (0.08, 0.09) | 19.70  (18.93, 20.49) |  | 0.00  (0.00, 0.00) | 5.57  (3.82, 7.87) |  | -0.03  (-0.05, -0.02) |  | -0.13  (-0.14, -0.11) | |
| Somalia | 0.63  (0.57, 0.68) | 16.71  (15.77, 17.63) |  | 0.02  (0.01, 0.03) | 5.61  (3.88, 7.89) |  | 1.61  (1.44, 1.77) | 16.19  (15.12, 17.16) |  | 0.06  (0.04, 0.08) | 5.43  (3.73, 7.63) |  | -0.10  (-0.11, -0.08) |  | -0.11  (-0.12, -0.09) | |
| South Africa | 4.88  (4.62, 5.17) | 18.66  (17.83, 19.54) |  | 0.16  (0.11, 0.23) | 6.13  (4.20, 8.63) |  | 9.40  (8.97, 9.86) | 18.52  (17.72, 19.36) |  | 0.29  (0.20, 0.41) | 5.92  (4.06, 8.30) |  | -0.01  (-0.01, 0.00) |  | -0.19  (-0.21, -0.16) | |
| South Korea | 4.24  (4.00, 4.48) | 12.61  (11.95, 13.30) |  | 0.12  (0.08, 0.17) | 3.78  (2.60, 5.37) |  | 10.55  (10.00, 11.12) | 12.66  (12.03, 13.32) |  | 0.29  (0.19, 0.42) | 3.48  (2.35, 5.03) |  | -0.01  (-0.02, 0.00) |  | -0.32  (-0.35, -0.29) | |
| South Sudan | 0.60  (0.56, 0.63) | 17.86  (17.09, 18.65) |  | 0.02  (0.01, 0.03) | 5.44  (3.75, 7.68) |  | 0.95  (0.90, 1.01) | 17.44  (16.72, 18.20) |  | 0.03  (0.02, 0.04) | 5.12  (3.52, 7.24) |  | -0.07  (-0.08, -0.06) |  | -0.18  (-0.19, -0.16) | |
| Spain | 5.32  (5.02, 5.63) | 10.52  (9.95, 11.09) |  | 0.15  (0.10, 0.21) | 2.94  (2.00, 4.19) |  | 8.42  (7.93, 8.92) | 10.42  (9.86, 10.97) |  | 0.24  (0.16, 0.34) | 2.73  (1.82, 3.98) |  | -0.03  (-0.04, -0.03) |  | -0.19  (-0.21, -0.17) | |
| Sri Lanka | 2.73  (2.60, 2.87) | 20.41  (19.64, 21.28) |  | 0.08  (0.05, 0.11) | 6.00  (4.05, 8.60) |  | 5.01  (4.84, 5.20) | 20.22  (19.49, 20.98) |  | 0.13  (0.09, 0.19) | 5.55  (3.75, 7.93) |  | -0.02  (-0.03, -0.01) |  | -0.25  (-0.26, -0.23) | |
| Sudan | 1.29  (1.19, 1.38) | 12.14  (11.42, 12.87) |  | 0.06  (0.04, 0.08) | 5.00  (3.59, 6.85) |  | 2.54  (2.36, 2.71) | 11.40  (10.70, 12.08) |  | 0.09  (0.06, 0.13) | 4.02  (2.84, 5.58) |  | -0.21  (-0.21, -0.20) |  | -0.75  (-0.75, -0.74) | |
| Suriname | 0.06  (0.06, 0.06) | 20.84  (19.72, 22.03) |  | 0.00  (0.00, 0.00) | 5.37  (3.61, 7.82) |  | 0.13  (0.12, 0.13) | 20.70  (19.53, 21.89) |  | 0.00  (0.00, 0.00) | 5.09  (3.38, 7.39) |  | -0.01  (-0.01, -0.01) |  | -0.18  (-0.18, -0.18) | |
| Sweden | 1.12  (1.06, 1.18) | 8.80  (8.36, 9.23) |  | 0.03  (0.02, 0.04) | 2.04  (1.35, 3.04) |  | 1.46  (1.39, 1.53) | 8.75  (8.33, 9.19) |  | 0.03  (0.02, 0.05) | 1.96  (1.28, 2.93) |  | -0.04  (-0.05, -0.02) |  | -0.18  (-0.22, -0.15) | |
| Switzerland | 0.97  (0.92, 1.03) | 10.21  (9.65, 10.78) |  | 0.02  (0.01, 0.03) | 2.32  (1.52, 3.47) |  | 1.50  (1.41, 1.60) | 10.13  (9.57, 10.72) |  | 0.03  (0.02, 0.05) | 2.22  (1.44, 3.36) |  | -0.03  (-0.03, -0.03) |  | -0.09  (-0.12, -0.05) | |
| Syria | 0.75  (0.69, 0.80) | 12.08  (11.38, 12.76) |  | 0.03  (0.02, 0.04) | 4.71  (3.34, 6.51) |  | 1.42  (1.34, 1.51) | 11.22  (10.57, 11.91) |  | 0.05  (0.03, 0.06) | 3.79  (2.64, 5.31) |  | -0.25  (-0.26, -0.24) |  | -0.74  (-0.75, -0.73) | |
| Taiwan(Province of China) | 3.58  (3.44, 3.72) | 19.55  (18.87, 20.28) |  | 0.09  (0.06, 0.13) | 4.94  (3.28, 7.17) |  | 6.63  (6.43, 6.85) | 19.30  (18.63, 19.98) |  | 0.16  (0.11, 0.23) | 4.67  (3.10, 6.81) |  | 0.00  (-0.03, 0.03) |  | -0.17  (-0.21, -0.12) | |
| Tajikistan | 0.55  (0.52, 0.58) | 16.88  (16.09, 17.69) |  | 0.02  (0.01, 0.02) | 4.94  (3.36, 7.06) |  | 1.07  (1.01, 1.13) | 16.60  (15.84, 17.43) |  | 0.03  (0.02, 0.04) | 4.73  (3.21, 6.73) |  | -0.04  (-0.07, -0.02) |  | -0.13  (-0.14, -0.12) | |
| Tanzania | 2.35  (2.20, 2.50) | 16.59  (15.78, 17.35) |  | 0.07  (0.05, 0.10) | 4.89  (3.39, 6.83) |  | 5.49  (5.17, 5.83) | 16.49  (15.76, 17.23) |  | 0.16  (0.11, 0.22) | 4.62  (3.16, 6.47) |  | 0.00  (-0.01, 0.00) |  | -0.17  (-0.18, -0.16) | |
| Thailand | 8.96  (8.58, 9.37) | 20.05  (19.32, 20.84) |  | 0.25  (0.17, 0.35) | 5.61  (3.81, 8.00) |  | 18.84  (18.15, 19.56) | 19.88  (19.13, 20.63) |  | 0.48  (0.32, 0.69) | 5.24  (3.51, 7.53) |  | -0.02  (-0.03, 0.00) |  | -0.17  (-0.20, -0.15) | |
| Timor-Leste | 0.09  (0.09, 0.10) | 20.39  (19.59, 21.26) |  | 0.00  (0.00, 0.00) | 6.14  (4.18, 8.75) |  | 0.19  (0.18, 0.20) | 20.17  (19.42, 20.97) |  | 0.01  (0.00, 0.01) | 5.71  (3.88, 8.16) |  | -0.03  (-0.05, -0.01) |  | -0.28  (-0.30, -0.26) | |
| Togo | 0.32  (0.30, 0.35) | 17.08  (16.30, 17.88) |  | 0.01  (0.01, 0.02) | 5.43  (3.72, 7.66) |  | 0.86  (0.81, 0.92) | 16.78  (16.04, 17.57) |  | 0.03  (0.02, 0.04) | 5.21  (3.56, 7.33) |  | -0.05  (-0.07, -0.03) |  | -0.12  (-0.13, -0.10) | |
| Tokelau | 0.00  (0.00, 0.00) | 19.76  (19.03, 20.53) |  | 0.00  (0.00, 0.00) | 5.46  (3.71, 7.77) |  | 0.00  (0.00, 0.00) | 19.72  (19.01, 20.44) |  | 0.00  (0.00, 0.00) | 5.17  (3.48, 7.41) |  | 0.01  (-0.01, 0.02) |  | -0.17  (-0.19, -0.15) | |
| Tonga | 0.01  (0.01, 0.01) | 19.78  (19.03, 20.57) |  | 0.00  (0.00, 0.00) | 5.44  (3.67, 7.72) |  | 0.02  (0.02, 0.02) | 19.65  (18.93, 20.39) |  | 0.00  (0.00, 0.00) | 5.23  (3.52, 7.40) |  | -0.01  (-0.02, 0.00) |  | -0.11  (-0.13, -0.09) | |
| Trinidad and Tobago | 0.19  (0.18, 0.21) | 20.8  (19.67, 22.05) |  | 0.00  (0.00, 0.01) | 5.20  (3.46, 7.60) |  | 0.37  (0.35, 0.39) | 20.75  (19.58, 21.93) |  | 0.01  (0.01, 0.01) | 4.96  (3.28, 7.23) |  | 0.01  (0.00, 0.01)* |  | -0.15  (-0.16, -0.14) | |
| Tunisia | 0.66  (0.62, 0.71) | 12.07  (11.35, 12.72) |  | 0.03  (0.02, 0.04) | 4.61  (3.28, 6.40) |  | 1.43  (1.35, 1.51) | 11.38  (10.75, 12.01) |  | 0.05  (0.03, 0.06) | 3.76  (2.62, 5.28) |  | -0.21  (-0.21, -0.20) |  | -0.70  (-0.72, -0.67) | |
| Turkey | 4.81  (4.48, 5.10) | 12.08  (11.38, 12.74) |  | 0.18  (0.12, 0.25) | 4.53  (3.21, 6.31) |  | 10.15  (9.57, 10.75) | 11.40  (10.75, 12.05) |  | 0.32  (0.22, 0.45) | 3.69  (2.56, 5.16) |  | -0.21  (-0.22, -0.20) |  | -0.73  (-0.75, -0.71) | |
| Turkmenistan | 0.39  (0.37, 0.41) | 16.88  (16.11, 17.71) |  | 0.01  (0.01, 0.02) | 4.79  (3.23, 6.8) |  | 0.74  (0.70, 0.77) | 16.83  (16.08, 17.60) |  | 0.02  (0.01, 0.03) | 4.52  (3.04, 6.47) |  | 0.00  (-0.02, 0.02) |  | -0.20  (-0.22, -0.18) | |
| Tuvalu | 0.00  (0.00, 0.00) | 19.62  (18.87, 20.45) |  | 0.00  (0.00, 0.00) | 5.51  (3.77, 7.88) |  | 0.00  (0.00, 0.00) | 19.64  (18.93, 20.42) |  | 0.00  (0.00, 0.00) | 5.26  (3.57, 7.49) |  | 0.01  (-0.01, 0.02) |  | -0.12  (-0.14, -0.10) | |
| Uganda | 1.30  (1.20, 1.39) | 14.63  (13.92, 15.31) |  | 0.04  (0.03, 0.06) | 4.17  (2.87, 5.87) |  | 3.08  (2.88, 3.29) | 14.47  (13.83, 15.14) |  | 0.08  (0.06, 0.12) | 3.78  (2.57, 5.38) |  | -0.05  (-0.05, -0.04) |  | -0.39  (-0.42, -0.37) | |
| UK | 10.98  (10.31, 11.64) | 13.39  (12.63, 14.15) |  | 0.29  (0.20, 0.43) | 3.57  (2.38, 5.18) |  | 12.53  (11.86, 13.26) | 12.09  (11.48, 12.74) |  | 0.35  (0.24, 0.50) | 3.26  (2.21, 4.73) |  | -0.49  (-0.59, -0.39) |  | -0.31  (-0.44, -0.18) | |
| Ukraine | 11.26  (10.76, 11.81) | 16.95  (16.22, 17.78) |  | 0.31  (0.21, 0.45) | 4.83  (3.28, 6.89) |  | 11.30  (10.78, 11.86) | 16.78  (16.03, 17.58) |  | 0.31  (0.21, 0.45) | 4.68  (3.18, 6.73) |  | -0.03  (-0.04, -0.02) |  | -0.09  (-0.09, -0.08) | |
| United Arab Emirates | 0.12  (0.11, 0.13) | 12.59  (11.91, 13.25) |  | 0.00  (0.00, 0.01) | 4.39  (3.09, 6.10) |  | 0.87  (0.80, 0.94) | 11.75  (11.12, 12.37) |  | 0.02  (0.01, 0.03) | 3.55  (2.46, 5.04) |  | -0.25  (-0.26, -0.24) |  | -0.74  (-0.78, -0.69) | |
| Uruguay | 0.46  (0.43, 0.49) | 12.45  (11.81, 13.10) |  | 0.01  (0.01, 0.02) | 3.98  (2.75, 5.63) |  | 0.60  (0.57, 0.63) | 12.31  (11.68, 12.95) |  | 0.02  (0.01, 0.03) | 3.70  (2.54, 5.26) |  | -0.04  (-0.05, -0.04) |  | -0.23  (-0.24, -0.22) | |
| USA | 42.11  (39.75, 44.7) | 13.79  (13.02, 14.58) |  | 1.34  (0.93, 1.87) | 4.34  (3.00, 6.06) |  | 70.71  (66.36, 75.15) | 14.00  (13.20, 14.83) |  | 2.19  (1.52, 3.05) | 4.28  (2.96, 5.99) |  | 0.03  (0.00, 0.05)* |  | -0.06  (-0.11, 0.00) | |
| Uzbekistan | 2.22  (2.10, 2.33) | 16.76  (15.99, 17.57) |  | 0.07  (0.05, 0.09) | 4.87  (3.30, 6.90) |  | 4.30  (4.08, 4.53) | 16.69  (15.95, 17.49) |  | 0.11  (0.07, 0.16) | 4.59  (3.11, 6.54) |  | -0.01  (-0.02, 0.00) |  | -0.19  (-0.21, -0.17) | |
| Vanuatu | 0.02  (0.02, 0.02) | 19.92  (19.16, 20.72) |  | 0.00  (0.00, 0.00) | 5.65  (3.86, 8.04) |  | 0.04  (0.04, 0.04) | 19.70  (18.96, 20.49) |  | 0.00  (0.00, 0.00) | 5.44  (3.67, 7.71) |  | -0.03  (-0.04, -0.01) |  | -0.12  (-0.13, -0.11) | |
| Venezuela | 2.49  (2.36, 2.64) | 20.77  (19.65, 21.99) |  | 0.06  (0.04, 0.09) | 5.24  (3.50, 7.60) |  | 6.13  (5.80, 6.49) | 20.54  (19.44, 21.75) |  | 0.15  (0.10, 0.22) | 5.02  (3.35, 7.42) |  | -0.02  (-0.03, -0.02) |  | -0.12  (-0.14, -0.10) | |
| Vietnam | 9.43  (8.95, 9.91) | 19.88  (19.06, 20.69) |  | 0.28  (0.19, 0.41) | 5.79  (3.94, 8.26) |  | 20.01  (19.24, 20.79) | 19.79  (19.04, 20.53) |  | 0.51  (0.34, 0.74) | 5.28  (3.55, 7.61) |  | 0.00  (-0.02, 0.01) |  | -0.28  (-0.30, -0.26) | |
| Virgin Islands US | 0.02  (0.02, 0.02) | 20.84  (19.63, 22.03) |  | 0.00  (0.00, 0.00) | 5.18  (3.44, 7.59) |  | 0.03  (0.03, 0.04) | 20.71  (19.54, 21.92) |  | 0.00  (0.00, 0.00) | 4.87  (3.22, 7.16) |  | -0.02  (-0.03, -0.01) |  | -0.21  (-0.22, -0.20) | |
| Yemen | 0.74  (0.68, 0.80) | 12.35  (11.62, 13.06) |  | 0.03  (0.02, 0.05) | 5.13  (3.63, 6.99) |  | 1.82  (1.67, 1.95) | 11.31  (10.63, 12.00) |  | 0.07  (0.05, 0.09) | 4.12  (2.92, 5.71) |  | -0.30  (-0.31, -0.29) |  | -0.80  (-0.83, -0.78) | |
| Zambia | 0.71  (0.67, 0.76) | 17.35  (16.55, 18.16) |  | 0.02  (0.02, 0.03) | 5.35  (3.63, 7.54) |  | 1.74  (1.64, 1.85) | 17.06  (16.34, 17.83) |  | 0.05  (0.03, 0.07) | 4.92  (3.34, 6.99) |  | -0.03  (-0.04, -0.02) |  | -0.24  (-0.26, -0.22) | |
| Zimbabwe | 0.94  (0.89, 1.00) | 17.03  (16.25, 17.82) |  | 0.03  (0.02, 0.04) | 5.15  (3.53, 7.26) |  | 1.55  (1.46, 1.65) | 16.73  (15.96, 17.53) |  | 0.05  (0.03, 0.06) | 4.94  (3.38, 6.92) |  | -0.07  (-0.08, -0.06) |  | -0.08  (-0.10, -0.05) | |
