# Supplementary material for: Global, regional, and national burden of age-related hearing loss from 1990 to 2019
Source: Aging (Albany NY). 2021 Dec 15;13(24):25944–59. doi: 10.18632/aging.203782 (PMC8751586; doi:10.18632/aging.203782)
Supplement: Supplementary Table 2 [file aging-13-203782-s003.pdf]

SUPPLEMENTARY TABLE

Supplementary Table 2. Age−related hearing loss prevalent cases and burden in 1990 and 2019 and the temporal trends from 1990 to 2019 in GBD regions.

Characteristics	1990				2019				EAPC (1990–2019)	
	Prevalent cases		DALYs		Prevalent cases		DALYs		ASPR	ASDR
		ASPR		ASDR		ASPR		ASDR		
	No. × 10^6 (95% UI)	No. × 10^{-2} (95% UI)	No. × 10^6 (95% UI)	No. × 10^{-3} (95% UI)	No. × 10^6 (95% UI)	No. × 10^{-2} (95% UI)	No. × 10^6 (95% UI)	No. × 10^{-3} (95% UI)	No. (95% CI)	No. (95% CI)
**GBD region**										
High−income Asia Pacific	25.70 (24.43, 27.01)	12.90 (12.28, 13.55)	0.75 (0.50, 1.07)	3.84 (2.60,5.46)	46.92 (44.34,49.52)	12.81 (12.18,13.45)	1.42 (0.97,2.01)	3.62 (2.46,5.19)	−0.03 (−0.03,−0.02)	−0.17 (−0.19,−0.15)
High−income North America	45.79 (43.22, 48.49)	13.58 (12.85, 14.35)	1.45 (1.01, 2.02)	4.26 (2.94, 5.96)	77.49 (73.00, 82.00)	13.71 (12.96, 14.48)	2.39 (1.67, 3.34)	4.18 (2.88, 5.85)	0.00 (−0.02, 0.03)	−0.08 (−0.13, −0.04)
Western Europe	57.52 (54.41, 60.78)	10.85 (10.30, 11.40)	1.54 (1.04, 2.23)	2.87 (1.95, 4.17)	79.97 (75.45, 84.83)	10.59 (10.04, 11.15)	2.16 (1.45, 3.12)	2.69 (1.81, 3.92)	−0.10 (−0.11, −0.09)	−0.16 (−0.18, −0.13)
Australasia	3.03 (2.92, 3.13)	13.19 (12.71, 13.64)	0.09 (0.06, 0.12)	3.83 (2.65, 5.43)	5.74 (5.40, 6.09)	13.01 (12.33, 13.77)	0.16 (0.11, 0.23)	3.57 (2.41, 5.10)	−0.03 (−0.05, 0.00)	−0.15 (−0.22, −0.09)
Andean Latin America	23.12 (21.95, 24.37)	21.24 (20.21, 22.34)	0.59 (0.40, 0.87)	5.61 (3.75, 8.11)	45.73 (43.90, 47.67)	18.58 (17.83, 19.35)	1.13 (0.74, 1.63)	4.67 (3.08, 6.74)	−0.20 (−0.29, −0.11)	−0.43 (−0.55, −0.32)
Tropical Latin America	5.05 (4.76, 5.35)	20.73 (19.52, 22.00)	0.13 (0.09, 0.19)	5.30 (3.56, 7.65)	12.17 (11.48, 12.90)	20.66 (19.48, 21.92)	0.29 (0.20, 0.43)	5.08 (3.41, 7.38)	0.00 (−0.01, 0.01)	−0.11 (−0.13, −0.09)
Central Latin America	21.32 (20.16, 22.53)	20.83 (19.76, 22.00)	0.54 (0.36, 0.79)	5.37 (3.61, 7.69)	50.72 (48.03, 53.58)	20.65 (19.56, 21.82)	1.23 (0.82, 1.80)	5.11 (3.42, 7.41)	−0.03 (−0.03, −0.03)	−0.17 (−0.18, −0.16)
Southern Latin America	5.77 (5.47, 6.10)	12.44 (11.80, 13.12)	0.18 (0.13, 0.26)	3.95 (2.73, 5.59)	9.84 (9.32, 10.38)	12.30 (11.66, 12.94)	0.30 (0.20, 0.42)	3.67 (2.52, 5.21)	−0.04 (−0.04, −0.03)	−0.23 (−0.24, −0.22)
Caribbean	5.83 (5.52, 6.16)	20.74 (19.64, 21.93)	0.15 (0.10, 0.22)	5.35 (3.57, 7.72)	10.52 (9.93, 11.11)	20.56 (19.42, 21.72)	0.26 (0.17, 0.37)	5.10 (3.40, 7.33)	−0.02 (−0.03, −0.02)	−0.15 (−0.16, −0.15)
Eastern Europe	45.30 (43.28, 47.37)	16.97 (16.24, 17.75)	1.24 (0.84, 1.76)	4.79 (3.25, 6.82)	51.89 (49.58, 54.25)	16.84 (16.12, 17.61)	1.40 (0.95, 2.00)	4.60 (3.13, 6.54)	−0.02 (−0.03, −0.01)	−0.11 (−0.12, −0.10)
Central Europe	23.90 (22.84, 25.01)	16.87 (16.15, 17.65)	0.64 (0.43, 0.91)	4.62 (3.11, 6.57)	30.42 (29.04, 31.95)	16.85 (16.12, 17.61)	0.81 (0.55, 1.16)	4.43 (3.00, 6.35)	0.00 (0.00, 0.01)	−0.11 (−0.13, −0.10)
Central Asia	8.67 (8.25, 9.09)	16.79 (16.04, 17.58)	0.25 (0.17, 0.35)	4.75 (3.21, 6.77)	13.74 (13.10, 14.40)	16.71 (15.98, 17.47)	0.35 (0.24, 0.51)	4.55 (3.07, 6.47)	−0.01 (−0.02, 0.01)	−0.14 (−0.15, −0.13)
North Africa and Middle East	24.09 (22.55, 25.59)	12.25 (11.57, 12.91)	0.95 (0.66, 1.31)	4.73 (3.38, 6.55)	54.84 (51.73, 57.98)	11.47 (10.86, 12.12)	1.76 (1.21, 2.48)	3.84 (2.69, 5.36)	−0.22 (−0.23, −0.21)	−0.72 (−0.74, −0.71)
South Asia	133.59 (126.21, 141.38)	18.97 (18.14, 19.86)	4.05 (2.72, 5.74)	5.69 (3.90, 8.04)	297.91 (284.41, 311.83)	19.08 (18.30, 19.92)	8.44 (5.74, 12.04)	5.50 (3.79, 7.79)	−0.02 (−0.04, −0.01)	−0.21 (−0.25, −0.17)
Southeast Asia	65.68 (62.55, 68.83)	20.19 (19.42, 20.97)	1.95 (1.30, 2.78)	5.88 (4.03, 8.36)	134.00 (128.85, 139.12)	20.12 (19.39, 20.87)	3.55 (2.38, 5.11)	5.56 (3.77, 7.91)	0.01 (−0.01, 0.02)	−0.14 (−0.15, −0.12)
East Asia	206.40 (196.71, 215.86)	20.49 (19.58, 21.37)	5.76 (3.87, 8.24)	5.87 (4.02, 8.36)	420.07 (402.28, 438.67)	21.05 (20.19, 21.94)	10.80 (7.24, 15.54)	5.69 (3.88, 8.11)	0.10 (0.07, 0.12)*	−0.14 (−0.19, −0.10)
Oceania	0.81 (0.77, 0.85)	19.98 (19.23, 20.74)	0.02 (0.02, 0.03)	5.66 (3.83, 8.04)	1.86 (1.77, 1.95)	19.86 (19.11, 20.63)	0.05 (0.03, 0.07)	5.51 (3.76, 7.81)	−0.01 (−0.03, 0.00)	−0.06 (−0.08, −0.05)
Western Sub−Saharan Africa	20.23 (19.00, 21.58)	17.21 (16.46, 18.00)	0.73 (0.49, 1.03)	5.83 (3.99, 8.15)	47.21 (44.29, 50.33)	17.07 (16.37, 17.86)	1.66 (1.12, 2.34)	5.63 (3.85, 7.84)	−0.01 (−0.03, 0.01)	−0.14 (−0.17, −0.11)
Eastern Sub−Saharan Africa	17.97 (16.76, 19.31)	17.47 (16.60, 18.31)	0.61 (0.40, 0.86)	5.58 (3.82, 7.87)	40.91 (38.25, 43.77)	17.42 (16.61, 18.24)	1.29 (0.87, 1.84)	5.31 (3.65, 7.51)	0.00 (0.00, 0.01)	−0.15 (−0.17, −0.14)
Central Sub−Saharan Africa	5.35 (5.02, 5.68)	17.20 (16.44, 17.96)	0.18 (0.12, 0.25)	5.49 (3.78, 7.64)	12.73 (11.90, 13.52)	16.76 (15.99, 17.50)	0.40 (0.27, 0.57)	5.15 (3.56, 7.25)	−0.07 (−0.10, −0.05)	−0.18 (−0.20, −0.16)
Southern Sub−Saharan Africa	6.39 (6.05, 6.77)	18.28 (17.49, 19.12)	0.21 (0.14, 0.30)	5.92 (4.06, 8.30)	11.99 (11.43, 12.60)	18.13 (17.36, 18.93)	0.37 (0.25, 0.52)	5.69 (3.92, 7.99)	−0.02 (−0.02, −0.01)	−0.17 (−0.19, −0.16)
